# Neurocognitive and cerebellar function in ADHD, autism and spinocerebellar ataxia

**DOI:** 10.3389/fnsys.2023.1168666

**Published:** 2023-06-21

**Authors:** Maurizio Cundari, Susanna Vestberg, Peik Gustafsson, Sorina Gorcenco, Anders Rasmussen

**Affiliations:** ^1^Department of Experimental Medical Science, Faculty of Medicine, Lund University, Lund, Sweden; ^2^Unit of Neuropsychiatry, Hospital of Helsingborg, Helsingborg, Sweden; ^3^Unit of Neurology, Hospital of Helsingborg, Helsingborg, Sweden; ^4^Department of Psychology, Faculty of Social Science, Lund University, Lund, Sweden; ^5^Child and Adolescent Psychiatry, Department of Clinical Sciences Lund, Medical Faculty, Lund University, Lund, Sweden; ^6^Department for Clinical Sciences Lund, Neurology, Lund University, Skåne University Hospital, Lund, Sweden

**Keywords:** cerebellum, cognition, neuropsychology, neurology, neuropsychiatry, spinocerebellar ataxia (SCA), attention deficit hyperactivity disorder (ADHD), autism spectrum disorder (ASD)

## Abstract

The cerebellum plays a major role in balance, motor control and sensorimotor integration, but also in cognition, language, and emotional regulation. Several neuropsychiatric disorders such as attention deficit-hyperactivity disorder (ADHD), autism spectrum disorder (ASD), as well as neurological diseases such as spinocerebellar ataxia type 3 (SCA3) are associated with differences in cerebellar function. Morphological abnormalities in different cerebellar subregions produce distinct behavioral symptoms related to the functional disruption of specific cerebro-cerebellar circuits. The specific contribution of the cerebellum to typical development may therefore involve the optimization of the structure and function of cerebro-cerebellar circuits underlying skill acquisition in multiple domains. Here, we review cerebellar structural and functional differences between healthy and patients with ADHD, ASD, and SCA3, and explore how disruption of cerebellar networks affects the neurocognitive functions in these conditions. We discuss how cerebellar computations contribute to performance on cognitive and motor tasks and how cerebellar signals are interfaced with signals from other brain regions during normal and dysfunctional behavior. We conclude that the cerebellum plays a role in many cognitive functions. Still, more clinical studies with the support of neuroimaging are needed to clarify the cerebellum’s role in normal and dysfunctional behavior and cognitive functioning.

## Introduction

Until recently, the cerebellum was regarded as a brain structure engaged mainly in motor control. Studies dating back to the early 19*^th^* century showed that cerebellar damage in pigeons resulted in an inability to fly due to uncoordinated wing movements (Flourens, 1824). After the cerebellum’s role in motor control was confirmed in studies on monkeys, it became a fundamental truth in clinical neurology and brain sciences (Russell and Horsley, 1894). Since then, numerous studies from the 20*^th^* and 21*^st^* centuries have reaffirmed the cerebellum as a structure that plays a vital role in motor control and motor learning (Manto et al., 2012). However, though the fields of cognitive neuroscience and experimental neuropsychology did not emerge until the early 20*^th^* century, other researchers in the 19*^th^* century reported emotional and intellectual impairment in patients with cerebellar agenesis (Fisher, 1839). Yet, it wasn’t until the 1990s that researchers refocused on the cerebellum’s non-motor functions (Schmahmann, 1991). Studies on cerebellar patients revealed significant impairments in executive function and attention (Stoodley and Schmahmann, 2010). Cerebellar abnormalities also have effects on other neurocognitive domains. These include language, visuospatial perception, visual construction ability, episodic memory, and social cognition (Hoche et al., 2016; Mariën and Borgatti, 2018; Slapik et al., 2019; Ray et al., 2020).

In line with these clinical findings, neuroimaging studies reveal that the cerebellum is anatomically and functionally interconnected with areas of the brain that are important for cognitive and emotional functions (Stoodley and Schmahmann, 2010). There is an abundance of mutual connections between the cerebellum and other areas of the brain, including the prefrontal cortex, the posterior parietal cortex, and the basal ganglia (Middleton and Strick, 1994; Kelly and Strick, 2003; Bostan et al., 2013).

This can explain why disorders affecting the cerebellum also lead to disrupted affective and cognitive functions, such as the cerebellar cognitive affective syndrome (CCAS) (Schmahmann and Sherman, 1998; Stoodley and Schmahmann, 2010; Ahmadian et al., 2019). Moreover, the profile and severity of CCAS symptoms varies in a predictable pattern depending on the pathological process and what part of the cerebellum is affected (Argyropoulos et al., 2020).

Here, we review literature on the anatomy and physiology of the cerebellum, and the neurocognitive functions of three conditions that have been linked to cerebellar function: SCA3, ADHD, and ASD. SCA3 is a neurological disease where cerebellar degeneration is clearly and robustly demonstrated, ADHD and ASD are neuropsychiatric disorders that have been associated with cerebellar abnormalities, but the extent of cerebellar involvement is not as pronounced as in the case of SCA3 (Riva et al., 2013; Wan et al., 2020; Duan et al., 2021; Stezin et al., 2021). We selected these three conditions to highlight the cerebellum’s role in cognition and how cognitive effects vary according to the severity of cerebellar abnormalities, more evident in SCA3 and lighter in ADHD and ASD.

We attempt to link these findings to contemporary theories and models of the cerebellum, and we discuss how symptoms may arise from interactions between the cerebellum and other brain parts that influence cognitive functions. We then discuss potential clinical implications from knowledge about the cerebellum’s involvement in cognitive functions. A cerebellar-dependent neuropsychological test battery could be a valuable tool in the diagnostic process when data from medical examinations such as biomarkers and neuroimaging data are missing or are unclear. This could lead to an improved understanding of the neurocognitive functions of these patient groups, and, ultimately, better treatment.

## Methods

### Search strategy and study selection

We performed a literature search in two electronic databases: PubMed and Embase. Through these searches, we identified articles supplying information on the neurocognitive profile, neurocognitive functions, and cerebellar functions of patients with three specific diagnoses ADHD, ASD, and SCA3.

Original articles and chapters reporting relevant studies on humans and animals were included. We used terms such as “ADHD,” “ASD,” and “SCA3” coupled with terms related to the cerebellum, cerebellar functions and to neurocognitive domains and neurocognitive profiles, such as “cognition,” “intelligence,” “visual perception/construction,” “processing speed,” “sensorimotor integration,” “executive function,” and “memory.”

### Cerebellum

The cerebellum (literally “little brain” in Latin) contains 80,2% of all neurons in the human brain as well as 19% of all the non-neural cells in the brain (Azevedo et al., 2009). The anterior lobe of the cerebellum receives afferents from the spinal cord through the spinocerebellar tracts and from the cerebral cortex *via* corticopontine projections (Brodal, 1978; Hartmann-von Monakow et al., 1981; Schmahmann et al., 2004). The posterior lobe receives input mainly from the brainstem and cerebral cortex (Siegel and Sapru, 2006). The cerebellar vermis and fastigial nucleus are interconnected with the vestibular nuclei and other brainstem nuclei engaged in motor control, gait, and equilibrium, and with brainstem nuclei linked with limbic and paralimbic cortical and subcortical regions (Voogd, 2004).

The cerebellum is not only connected with structures concerned with motor control. Large parts of the cerebellum forms reciprocal connections with association areas of the cerebral cortex including the prefrontal cortex, posterior parietal cortex, superior temporal polymodal regions, cingulate gyrus, and posterior parahippocampal area (Strick et al., 2009; Bostan et al., 2013). The anatomical circuits linking the cerebellum with the cerebral cortex are organized in a two-stage feed-forward loop (the corticopontine and pontocerebellar projection) and a two-stage feedback loop (cerebellothalamic-thalamocortical projection). These loops are constituted by multiple parallel but partially overlapping subcircuits that link different areas of the cerebral cortex with specific regions of the cerebellum (Brodal, 1978; Voogd and Glickstein, 1998; Voogd, 2004; Apps and Garwicz, 2005). The cerebellum is also mutually interconnected with the basal ganglia *via* oligosynaptic loops (Hintzen et al., 2018). These anatomical connections hint that the cerebellum plays a role that goes beyond motor control. Indeed, as we will discuss below, evidence from recent decades suggests that the cerebellum is highly involved in a wide spectrum of cognitive functions.

Output from the cerebellar cortex goes *via* the cerebellar nuclei. The medial (fastigial) nucleus projects mainly to the brainstem and spinal cord; the interposed (comprising the emboliform and globose) nucleus targets mainly the midbrain and the thalamus; and the lateral (dentate) nucleus projects mainly to the thalamus. Some neurons in the cerebellar nuclei project directly to the red nucleus, which then projects to other nuclei that control muscles. This is how the cerebellum can elicit blink responses even in the absence of the cortex (Hesslow and Yeo, 2002). More recently, it was discovered that the cerebellar nuclei also contain neurons that project to the Purkinje cells forming the nucleo-cortical cerebellar pathway (Ankri et al., 2015). Apart from the inhibitory input from the Purkinje cells, the cerebellar nuclei also receive excitatory input from collaterals of both mossy fibers and climbing fibers (Ten Brinke et al., 2017).

#### Functions of the cerebellum

Knowledge of cerebellar functions has been derived mostly from studying natural and experimental ablative lesions (Fine et al., 2002). The cerebellum plays a crucial role in making predictions and creating feedforward models that guide our behavior (Wolpert et al., 1998; Welniarz et al., 2021). Consequently, cerebellar damage makes movements jerky, slow, and uncoordinated–as is evident in ataxia and dystonia (Therrien and Bastian, 2015). In line with the cerebellum’s role in motor coordination, disruption of the ionic homeostasis in cerebellar neurons has been shown to evoke motor impairments in mouse model studies (Hoxha et al., 2018). The impairments range from gross abnormalities to more specific impairments. For example, disruption of the high-voltage activated Ca^2+^-influx in Purkinje cells severely disrupts action potential firing and motor behavior in mouse model studies (Tara et al., 2018). Likewise, deletion of several chloride channels and transporters in mouse models has been associated with motor abnormalities (Poroca et al., 2017). For instance, knockout mouse models with deletion of GABA_*A*_ receptors from Purkinje cells did not show ataxia, but they displayed significant impairments in several aspects of locomotion, motor performance and motor learning (Wulff et al., 2009).

Clinical research from the last three decades show that the cerebellum is not merely concerned with motor functions. The human cerebellum is linked with cerebral association areas concerned with intellect, social cognition, perception, recognition, forwarding and encoding of emotional information, and the regulation of emotional states in relation to cognitive, motor and social behaviors (Stoodley and Schmahmann, 2009a,b, Stoodley et al., 2010; Adamaszek et al., 2017). In line with the anatomical connections between the cerebellum, the basal ganglia (K B et al., 2022), and the cerebral cortex (Allen and Tsukahara, 1974; Holdefer et al., 2000; Strick et al., 2009), the cerebellum has been shown to play a role in many cognitive and emotional functions (Reeber et al., 2013). The limbic/cognitive cerebellum is identified in the cerebellar posterior lobe; but also, others areas of the cerebellum contribute to cognition like the sensorimotor cerebellum represented in the anterior lobe (Schmahmann, 2019). Furthermore, cerebellar damage is associated with lesions in the posterior lobe resulting in deficits in executive functions, visuospatial processing, language abilities and affect regulation (Karatekin et al., 2000; Grill et al., 2004; Manes et al., 2009). Other disorders linked to morphological abnormalities in the cerebellum, such as ASD, schizophrenia, and ADHD have also been linked with affective dysregulation (Bernard and Mittal, 2014; D’Mello et al., 2015; Stoodley, 2016; Kim et al., 2021). The cerebellum also contributes to visual cognition. Patients with degenerative cerebellar disorders showed impairments on mental rotation, an operation that requires continuous manipulation of a visual representation (McDougle et al., 2022). Further, the cerebellum is involved in face and emotion processing *via* its connections with limbic and cortical areas important for processing facial emotions perception (Fusar-Poli et al., 2009; Schutter et al., 2009). The cerebellum is involved as well in timing (Ivry and Keele, 1989).

#### Neuroimaging, cognitive and behavioral findings

Neuroimaging studies have verified that the cerebellum contributes to a broad range of cognitive functions, like perception, language, working memory, cognitive control, and social cognition (Diedrichsen et al., 2019). A review of clinical, experimental and neuroimaging studies shows that sensorimotor tasks have been linked to the anterior lobe, while higher function tasks, including language, executive function, and working memory, have been linked to the posterior lobe of the cerebellum. Processing of emotions was localized to vermal lobule VII and lateral posterior hemisphere (Stoodley and Schmahmann, 2009a). The involvement of the cerebellum in these processes is due to functional connections with frontal lobes *via* the cerebello-thalamo-cortical and cortico-ponto-cerebellar loops (Middleton and Strick, 1994; Kelly and Strick, 2003).

Functional magnetic resonance imaging (fMRI) shows cerebellar activation during many cognitive, affectional, and social tasks in healthy participants, although there is significant inter-participant variability in the pattern of topographical activation on a specific task (King et al., 2019). A meta-analysis concluded that specific parts of the cerebellum were activated during cognitive tasks and music elaboration/processing (E et al., 2014). One of the first positron emission tomography (PET) studies examining the cerebellum by Petersen et al. (1988) showed activation in the right lateral cerebellum when participants generated words. The cerebellum is also involved in lexical retrieval, syntax, aphasia, reading, writing and metalinguistic skills (De Smet et al., 2013). Other cognitive functions like working memory, social cognition, emotional memory, semantic memory and visuospatial abilities are also linked to the cerebellum (Ashida et al., 2019; Slapik et al., 2019; Van Overwalle et al., 2020; Gatti et al., 2021; Fastenrath et al., 2022). Another fMRI study showed that the dentate, the output nucleus of the cerebellum, was activated by cognitive processing, for example, during a realization of a puzzle that required multiple cognitive processes (Kim et al., 1994).

Petacchi et al. (2005) analyzed studies that used fMRI and PET techniques, their analysis shows that specific regions in the cerebellum are activated during auditory tasks and contribute to sensory auditory processing. Recent results demonstrated that the cerebellum shows increased activity during enhanced emotional memory encoding; the midline cerebellum seems to be activated during recall of emotional personal life episodes, remarking an aspect of this region in emotional memory retrieval (Fastenrath et al., 2022).

Neuroimaging studies have shown that explicit motor timing activates the cerebellum, the supplementary motor area, basal ganglia, right-inferior frontal, and parietal cortices (Avanzino et al., 2016). Timing ability affects sequential rhythmic movements, motor control, and many cognitive tasks used to assess executive functions (Homack et al., 2005). The cerebellum is essential in timing-dependent learning processes. It plays a role in finger tapping, in which the participant must maintain a rhythm, and eyeblink conditioning, a simple form of associative motor learning in which subjects learn to associate a neutral stimulus with a reflex-eliciting stimulus (Gerwig et al., 2007; Bracha et al., 2009). The cerebellum, together with other brain areas like the primary sensorimotor cortex, the supplementary motor area, and the occipital cortices, are necessary to complete the finger-tapping task in response to visual cues (Turesky et al., 2018). Previous studies have brought to light that the main neuronal plasticity responsible for generating the conditioned eyeblink response takes place in specific regions of the cerebellum (Steinmetz, 2000; Thürling et al., 2015).

#### Spinocerebellar ataxia type 3 (SCA3)

Ataxia derives from Greek and means “lack of order.” SCA is a rare inherited (autosomal dominant), progressive, neurodegenerative, and heterogeneous disease mainly affecting the cerebellum. SCA is the used nomenclature for Spinocerebellar Ataxia from type 1 to type 48 for autosomal dominant cerebellar ataxia a (Kuo, 2019). SCA3 is the world’s most common, dominantly inherited ataxia. Ataxia is characterized by decreased voluntary muscle coordination and impairment of movement control that affects gait stability, speech, and eye movement. Ataxia refers to the pattern of impaired movement and the group of diseases characterized by this specific symptom. The primary manifestation is losing balance while standing and walking (Ashizawa and Xia, 2016). Ataxia can be inherited, acquired, or idiopathic. Based on the inheritance mode, cerebellar ataxias are classified as Autosomal Dominant Cerebellar Ataxias, also known as SCA, Autosomal Recessive Cerebellar Ataxias, mitochondrial or X-linked ataxias (Durr, 2010; Klockgether, 2010). Acquired ataxias can be caused by many factors, such as strokes, tumors, multiple sclerosis (Kuo, 2019), alcoholism, peripheral neuropathy, metabolic disorders, or vitamin deficiencies (Klockgether, 2010). Cerebellar lesions associated with ataxia are often seen in neuroimaging (Kuo, 2019).

Due to the effects on the cerebellum, CCAS is present in SCA3 (Paulson, 2012). However, CCAS has been studied in SCA3 (Braga-Neto et al., 2012; Maas et al., 2021). Dystonia, depression, diplopia, sensorimotor polyneuropathy, restless legs syndrome, and parkinsonism are the recognized clinical symptoms of SCA3 patients (Kuo, 2019). Several genetic causes exist for ataxia; the CAG-repeat expansion is the leading cause (Durr, 2010). Besides the effect on the cerebellum (see below), the connectivity is impaired in both the striatal-cortical and cerebellar-cerebral networks (Lindsay and Storey, 2017). The striatal-cortical network, exactly the prefrontal striatal circuit, sustains a decisive role in higher-order cognitive functions (Alexander et al., 1986).

#### Cerebellum and ataxia

SCA3 influences the brain stem, cerebellum, and pyramidal tracts (D’Abreu et al., 2012; Wu et al., 2017). However, other studies also show an effect on other brain regions, such as basal ganglia, pallidum, and diencephalon (Arruda et al., 2020). Morphometric MRI has demonstrated typical patterns of atrophy or volume loss in the cerebellum and brainstem with ulterior lesions in some supratentorial areas (Wan et al., 2020; Stezin et al., 2021; van der Horn et al., 2022; Yap et al., 2022a). Some specific focal or regional atrophies noted in certain SCAs are present in SCA3, where studies show pontocerebellar atrophy with enlargement of the fourth ventricle (Bhandari et al., 2022). Animal models helped to clarify the pathogenesis of the cerebellar ataxias (Burright et al., 1995; Serra et al., 2006). Purkinje cell degeneration is one of the primary mechanisms in animal models of ataxia (Chen et al., 2008; Liu et al., 2009). Data from calcium imaging and electrophysiological studies demonstrate that Purkinje cells and their significant inputs are malfunctioning before the onset of degeneration in ataxia mouse models (Barnes et al., 2011; Hourez et al., 2011; Shakkottai et al., 2011; Kasumu et al., 2012; Hansen et al., 2013). Alterations of cerebellar function can be identified with motor learning paradigms that depend on the integrity of the olivocerebellar circuit, such as eyeblink. A recent study shows that this type of learning is impaired in preclinical carriers of a SCA3 mutation (van Gaalen et al., 2019).

The cerebellum has enduring subcortical and cortical connectivity, resulting in non-motor symptoms that are often neglected during clinical assessments in many neurodegenerative conditions, especially if clinical ataxia is not present (Schmahmann et al., 2004; Moro et al., 2019; Fastenrath et al., 2022). More than forty SCA have been recognized, SCA does not affect only the cerebellum, and SCA3 may involve other areas of the central nervous system, like pontine nuclei, spinal cord, cortex, peripheral nerves, and basal ganglia (Bhandari et al., 2022). Neural functions can be altered even if there are no visible imaging results, and these can be the primary cause of behavioral symptoms in cerebellar ataxia. Furthermore, cerebellar gray matter atrophy is found in several other neurodegenerative diseases such as amyotrophic lateral sclerosis, Alzheimer’s disease, frontotemporal dementia, Huntington’s disease, multiple systemic atrophy, Parkinson’s disease, and progressive supranuclear palsy (Gellersen et al., 2017). These studies together highlight the cerebellum’s role in both motor and cognitive functions, and they suggest that SCA affects motor function and cognition.

#### Neurocognitive functions in spinocerebellar ataxia type 3

Although previously thought to primarily impact motor functions, numerous studies that we review below highlight that SCA3 also significantly impacts multiple cognitive domains.

#### Executive functions

Previous studies show that SCA3 patients exhibit deficits on the Hayling Sentence Completion Test and the Stroop Color-Word Test–designed to assess inhibitory ability (Zawacki et al., 2002; Garrard et al., 2008; Moriarty et al., 2016). Feng et al. (2014) showed that the time required to complete Trail Making Test A did not differ significantly between SCA3 patients and controls, but it took significantly more time for SCA3 patients to finish Trail Making Test B. Results from Braga-Neto et al. (2012) showed that both Trail-Making Tests A and B were significantly more impaired in patients with SCA3 than controls. Patients with SCA3 have some deficits in auditory working memory, as measured by the Backward subtest of the Digit Span Test (Feng et al., 2014; Ma et al., 2014; Tamura et al., 2018). Several studies did, however, show average performance on Digit Span Test Forward, a measure of short-term memory that is more related to attention than to working memory (Zawacki et al., 2002; Bürk et al., 2003; Kawai et al., 2004; Braga-Neto et al., 2012; Lopes et al., 2013; Feng et al., 2014). Deficits in visuospatial working memory were also detected using Spatial Span and Corsi-Block tapping test (Braga-Neto et al., 2012; Lopes et al., 2013). The executive function impairments may demonstrate an interrupted cortico-striatal and cerebro-cerebellar network in patients affected by SCA3, coherent with similar cognitive difficulties highlighted in CCAS (Stoodley and Schmahmann, 2010; Lindsay and Storey, 2017).

#### Attention and processing speed

Contrasting results emerged for this cognitive domain. Yap et al. (2022b) described no impairment, while other studies show that SCA is associated with impaired visuospatial attention and processing speed (Zawacki et al., 2002; Braga-Neto et al., 2012; Tamura et al., 2018). The grade of ataxia severity, visuomotor search and cerebellar oculomotor problems may interfere with performance on neurocognitive tests (Braga-Neto et al., 2012; Tamura et al., 2018). On the other hand, auditory attention is not impaired in SCA3 (Garrard et al., 2008). The Symbol Digit Modalities Test and Elevator Counting subtest of the Test of Everyday Attention show impairment related to attention and process speed (Moriarty et al., 2016).

#### Language

Given that the cerebellum is also involved in language, it is perhaps unsurprising that SCA is associated with language problems (Mariën and Borgatti, 2018). One study on twenty SCA3 patients demonstrated that semantic fluency, phonemic fluency, and category switching were significantly lower in SCA3 patients compared to healthy controls (Maas et al., 2021); the same results were found in a longitudinal study (Moriarty et al., 2016). Verbal fluency was assessed in letter and category fluency tasks, it was also found to be impaired in multiple studies (Kawai et al., 2004; Klinke et al., 2010; Lopes et al., 2013; Feng et al., 2014; Ma et al., 2014; Tamura et al., 2018).

Besides, contrasting test results emerge for semantic verbal fluency with and without impairment (Bürk et al., 2003; Kawai et al., 2004; Garrard et al., 2008; Klinke et al., 2010; Braga-Neto et al., 2012; Feng et al., 2014; Tamura et al., 2018). According to some studies, the naming ability of SCA3 patients, an aspect of the semantic memory which requires retrieval capacity, is not impaired (Zawacki et al., 2002; Kawai et al., 2004; Lopes et al., 2013).

To some extent, impairment in executive functions may mediate the language deficits seen in patients with SCA. For example, patients may have trouble with word retrieval that implicates the cerebellum and its connections with the cerebrum (Mariën et al., 2014). Fundamental executive dysfunction in patients with SCA3 may interfere with the retrieval processes of verbal information from memory storage (Tamura et al., 2018). Divergent phonemic and semantic verbal fluency results indicate the cerebellum’s role in the motor and sequential speech mechanisms. Cerebellar impairment can affect verbal fluency by explicitly affecting the performance of phonemic rules. These results highlight the importance of computational properties in developing linguistic strategies by the cerebellum (Leggio et al., 2000).

#### Memory

Patients with SCA also exhibit memory problems. Several studies show that SCA3 patients have deficits with immediate auditory recall and delayed auditory recall (Bürk et al., 2003; Kawai et al., 2004; Garrard et al., 2008; Lopes et al., 2013; Feng et al., 2014; Ma et al., 2014; Tamura et al., 2018). A single study shows impairment of delayed recall but average immediate recall (Elyoseph et al., 2020). Deficits in both immediate and delayed recall in visual episodic memory were identified with the Visual Paired Associate test, and delayed recall in visual episodic memory was detected with the Visual Reproduction test. Scores were independent of ataxia severity even though motor coordination is necessary to complete this test (Kawai et al., 2004). Different results were obtained in another study that utilized the Visual Reproduction Test of Wechsler Memory Scale-III, where immediate visual recall was poorer while delayed visual recall was intact (Garrard et al., 2008).

SCA3 patients did not show any visual recall impairment measured with the Rey-Osterrieth Complex Figure test, a non-process-pure test that can measure more cognitive functions (Meyers and Kr, 1995; Bürk et al., 2003; Braga-Neto et al., 2012). Mainly, auditory and visual recognition memory was intact in several studies (Zawacki et al., 2002; Garrard et al., 2008; Klinke et al., 2010; Lopes et al., 2013; Elyoseph et al., 2020). A study that used the Recognition Memory Test showed that SCA3 patients have more impairment in face recognition than word recognition (Moriarty et al., 2016). Moreover, auditory and visual recognition is usually unaffected, indicating that encoding and consolidation work and memory difficulties result from impaired free recall. Problems with recall are due to impairments of executive functions. A study shows contrasting results for procedural learning, where SCA3 patients have impaired procedural learning of the sensory-motor sequence on the procedural Serial Reaction Time Test but regular learning on the procedural Weather Prediction Probabilistic Classification test (Elyoseph et al., 2020).

The results on sequence learning impairment are coherent with the present opinion on dysregulated striatal-cortical and cerebellar-cerebral networks in SCA3 (Lindsay and Storey, 2017). Parietal and prefrontal areas are activated during mindful learning and recall of sequence information mediated by working memory, where the cerebellum and motor-related areas are accountable for the habituation process (Eliassen et al., 2001). An impaired auditory and visual recall is present in SCA3 patients. Recognition memory is intact; cognitive impairments influence information retrieval, which is necessary for free recall test conditions. These study results do not prove a temporal lobe impairment (Lalonde and Botez-Marquard, 2000), where recall and recognition memory are significantly impaired. However, these results could confirm the involvement of a dysregulated prefrontal-striatal network (Lindsay and Storey, 2017). These results are coherent with the cognitive profile of the cerebellar cognitive affective syndrome, where the memory is altered as a consequence of impaired executive functions (Ray et al., 2020). In conclusion, several studies show that SCA3 is associated with memory deficits–especially for memory functions related to the frontal cortex.

#### Visuospatial perception and visuospatial ability

In contrast to the cognitive domains discussed so far, where there are evident deficits, at least in some sub-domains, the evidence for deficits in visuospatial perception and ability is relatively weak. Visual perception has been measured in SCA3 patients with the Incomplete Letters subtest of the Visual Object and Space Perception Battery. Results showed performances within the normal range (Garrard et al., 2008). No impairment was detected in studies that utilized the copy trial of the Rey–Osterrieth Complex Figure test, which measures visual-constructional ability and visual memory (Bürk et al., 2003; Braga-Neto et al., 2012). In contrast, the cube drawing test, which requires a three-dimensional perspective, showed impairments in this category of patients (Maas et al., 2021).

#### Social cognition

Theory of Mind is impaired in SCA3 patients (Moriarty et al., 2016). However, the ability to identify normative situations or violations of normative situations and attribute emotions like happiness, sadness, fear, anger or embarrassment is not impaired (Garrard et al., 2008; Sokolovsky et al., 2010). Difficulties emerged with the ability to describe, interpret, and justify the main character’s behavior during real social situations, presented in 14 stories. Social cognition has, however, rarely been examined in these patients, perhaps because SCA has always been treated as a disease that affects motor performance above other things. More research on the effects of cerebellar damage on social cognition is needed.

#### General intelligence

Several studies have investigated the impact of SCA3 on general intelligence, with contradictory results. A recent review by Yap et al. (2022b) concluded that general intelligence and neurocognitive functions such as verbal reasoning, semantic language, recognition, visuospatial perception, visuoconstructive ability, attention, and processing speed are relatively preserved in SCA3 patients (Yap et al., 2022b). Some studies reported the preservation of general intelligence, as measured by a composite score that includes both non-verbal and verbal reasoning (Zawacki et al., 2002; Bürk et al., 2003; Kawai et al., 2004; Kamphaus, 2005; Klinke et al., 2010), while one study underlined lower intelligence level compare to the control group (Garrard et al., 2008). However, one study reported lower general intelligence scores in SCA3 patients compared to the average population (Garrard et al., 2008). On the other hand, verbal reasoning appeared to be preserved, with average scores found on the Verbal Comprehension subtest of the Wechsler Adult Intelligence Scale (Zawacki et al., 2002; Bürk et al., 2003; Garrard et al., 2008; Klinke et al., 2010; Braga-Neto et al., 2012; Lopes et al., 2013). The results for non-verbal reasoning were less consistent with some studies demonstrating apparent impairment on the Perceptual Reasoning subtest of the WAIS (Kawai et al., 2004; Garrard et al., 2008; Braga-Neto et al., 2012), while others did not find any significant impairment (Zawacki et al., 2002; Bürk et al., 2003; Lopes et al., 2013). Overall, the literature suggests that general intelligence is, at most, only mildly impacted in SCA3 patients.

#### Summary

In conclusion, multiple studies on SCA3 patients with cerebellar damage have shown impairments in various cognitive domains, particularly in executive functions, and secondary effects in other areas. The neurocognitive functions are illustrated in Table 1. The cognitive profile of SCA3 shows impairment in the executive functions of inhibition, cognitive flexibility, and visual and verbal working memory (Zawacki et al., 2002; Braga-Neto et al., 2012; Feng et al., 2014).

**TABLE 1:** Summarizes the evidence in the papers reviewed.

Cognitive domain		SCA3	ADHD	ASD
Executive functions	Inhibition	↓↓	↓↓	↓
	Shifting	↓	↓↓	↓
	Auditory working memory	↓	↓⇄	↓↓
	Visuospatial working memory	↓	↓⇄	↓
Attention and processing speed	Complex attention	↓⇄	↓↓	↓
	Processing speed	↓⇄	↓↓	↓⇄
Language	Semantic fluency	↓⇄	⇄	↓
	Phonemic fluency	↓	⇄	↓
	Category switching	↓	⇄	↓
	Naming ability	↓	↓	↓
	Verbal intelligence quotient	↓⇄	↓	↓↓
Memory	Verbal immediate recall	↓⇄	↓	↓⇄
	Verbal delayed recall	↓↓	↓ ⇄	↓
	Verbal recognition	↓⇄	↓
	Visual immediate recall	↓	↓	↓
	Visual delayed recall	↓	↓	↓
	Visual recognition	⇄	↓
	Procedural memory	↓⇄	⇄	↓
Visuospatial perception and visuospatial ability	Visuospatial perception	↓⇄	↓⇄	⇄
	Visuospatial ability	↓⇄	↓⇄	⇄
	Perceptual quotient	↓⇄
Social cognition	Theory of mind	↓	↓⇄	↓↓

The symbols indicate to what extent SCA3, ADHD, and ASD are associated with deficits in different cognitive domains. The table also indicates whether there is a consensus or whether the papers reviewed have different findings. **↓↓** = strong evidence of impairment: (multiple studies converging on the same conclusion). **↓** = weak evidence of impairment (previous studies have low number of subjects and multiple confounders which effect the study results). **⇄** = contrasting results (results in previous studies show that cognitive domain was impaired in some studies but intact in other studies).

Previous results showed a link between activity in the cerebro-ponto-cerebello-thalamo-cortical loop and executive functions in SCAs (Lindsay and Storey, 2017). This is consistent with the previously reported core impairment of CCAS (Stoodley and Schmahmann, 2010; Lindsay and Storey, 2017).

Executive dysfunctions also affect verbal functions like semantic and phonemic fluency as well as fluency while switching between categories (Kawai et al., 2004; Moriarty et al., 2016; Maas et al., 2021). These are all functions that demand retrieval from long-term semantic memory. Semantic memory seems to be unaffected in these patients (Zawacki et al., 2002; Kawai et al., 2004; Lopes et al., 2013). Executive dysfunctions also impact episodic memory, with results showing evident deficits in information retrieval in visual and verbal episodic memory, while recognition and consolidation of information are partially intact (Bürk et al., 2003; Garrard et al., 2008; Braga-Neto et al., 2012; Moriarty et al., 2016). Visuospatial attention, visuospatial ability, and processing speed are also impaired (Zawacki et al., 2002; Moriarty et al., 2016; Tamura et al., 2018).

While social cognition is not well-studied in patients with SCA, some findings suggest that the Theory of Mind may be affected. Psychiatric conditions such as anxiety and depression, common among SCA3 patients, may also harm cognition. Language and cultural differences may also confound psychological studies, including those we have reviewed here. The severity of ataxia, the duration of the disease, and visual, articulatory, or upper limb motor difficulties can also influence cognition and hence the performance on neuropsychological assessments. However, because of the small sample sizes of previous studies, different confounders, and different neuropsychological methods that have been used, the neurocognitive profile of SCA3 needs to be better characterized and needs to be investigated further.

### Attention deficit hyperactivity disorder

ADHD is a neuropsychiatric disorder present in approximately 5% of all children and in approximately 2.5% of the adult population (Regier et al., 2013; Austerman, 2015). It is estimated that about 15% of patients in adult psychiatry have ADHD (Deberdt et al., 2015). ADHD is defined based on clinical symptoms indicating dysregulation of attention and or activity/impulse control. When ADHD is suspected in adulthood, the investigation can be supplemented with the Wender Utah Rating Scale and Adult ADHD Self-Report Scale (Anbarasan et al., 2020).

Adults with ADHD show emotional dysregulation and executive function deficits (Barkley, 2010; Kessler et al., 2010; Biederman et al., 2019). While adults with ADHD often find ways to deal with attention problems and hyperactivity, the demands on executive abilities increase. Most jobs require complex planning and organizational skills, causing difficulties for adults with ADHD. Organizational problems often cause frustration and despair that lead to anxiety, sleep disorders, fatigue, depression or other conditions (Brattberg, 2006; Dirks et al., 2017; Schiweck et al., 2021). ADHD symptoms are normally distributed in the population, meaning they are present to varying degrees in many people. It is only when the symptoms are prominent and create a significant functional impairment that an ADHD diagnosis is justified. To receive the diagnosis, manifestations of the main symptoms of inattention and/or hyperactivity and impulsivity, along with clear functional problems due to ADHD symptoms, should be present before the age of twelve (Doernberg and Hollander, 2016). Patients with ADHD do not just look for treatment and assistance for what is classically perceived as ADHD, but for various psychiatric symptoms and functional problems, such as depression, fluctuating mood, fatigue, and anxiety (Brattberg, 2006). In summary, to diagnose ADHD is still challenging. This heterogeneous neuropsychiatric condition has many comorbidities with similar phenotypes like anxiety disorders, mood disorders, bipolar disorder, hypomania, substance use disorders, sleep disorders, Tourette syndrome, ASD and other behavioral disorders (Hegerl et al., 2010; El Malhany et al., 2015; Doernberg and Hollander, 2016; Fadeuilhe et al., 2021; Schiweck et al., 2021). Even if ADHD is considered a neurodevelopmental disorder, ADHD symptoms usually persist in late adulthood and is often confounded with mild cognitive impairment (Callahan et al., 2017).

#### Cerebellum and ADHD

The brain of patients with ADHD has been the subject of extensive research. One of the most robust findings is the abnormal structure and function of the cerebellum in patients diagnosed with ADHD. Abnormal cerebellar development has been implicated in ADHD (Vloet et al., 2006; Vaidya, 2011; Stoodley, 2016; Bruchhage et al., 2018). Functional imaging studies point to abnormalities in the cerebellum and other brain structures. Cerebellar and cerebral volume is ∼4% smaller in ADHD patients compared to controls (Castellanos et al., 2002; Vaidya, 2011). One study which used diffusion tensor imaging showed that children with ADHD have thinner, less myelinated, and less consistently organized fibers in the right cerebral peduncle, left middle cerebellar peduncle, and left cerebellum (Ashtari et al., 2005). Previous studies using MRI in children with ADHD have observed structural changes in cerebellum and specifically in the vermis (Castellanos et al., 1996; Berquin et al., 1998; Mackie et al., 2007). A section of the executive/non-motor portion of the cerebellum, the left Crus I result has been shown to have a lower grey-matter volume in ADHD children compared to healthy controls (Fernandez et al., 2022).

However, the brain changes associated with ADHD extend beyond the cerebellum. Structural brain imaging studies have revealed that patients with ADHD display smaller brain volumes in multiple regions, including the cerebellum, dorsolateral prefrontal cortex, caudate, pallidum, and corpus callosum (Seidman et al., 2005; Luders et al., 2009). Moreover, morphological and functional imaging studies in patients with ADHD have identified abnormal white-matter structure of neural pathways connecting prefrontal and parieto-occipital areas with the striatum and the cerebellum (Silk et al., 2009).

This evidence of ADHD being linked to cerebellar abnormalities is further supported by studies revealing that children with ADHD perform poorly on tasks that demand cerebellar involvement. Children with ADHD exhibit abnormal tapping responses and abnormal synchronized oscillatory mechanisms (Rivkin et al., 2003; Ben-Pazi et al., 2006). Children with focal cerebellar disorders and ADHD showed assorted impairments of eyeblink conditioning (Frings et al., 2010; Reeb-Sutherland and Fox, 2015). Ample research shows that ADHD is associated with changes in cerebellar morphology and cerebellar function (Buderath et al., 2009; Frings et al., 2010; Bledsoe et al., 2011). The role of the vermis in ADHD is still not clear but some researchers have pointed out its possible role. Bledsoe et al. (2011) highlighted that a smaller volume of the posterior inferior vermis is connected to behavioral outcomes like hyperactivity, attention and impulsivity reported by parents of children with ADHD.

Increased cerebro-cerebellar functional connectivity in the superior temporal gyrus within the somatomotor network could underlie impairments in cognitive control and somatic motor function in ADHD. Furthermore, increasing cerebro-cerebellar functional connectivity in older participants with ADHD suggests that enhanced cerebellar involvement may compensate for dysfunctions of the cerebral cortex in ADHD (Wang et al., 2022). Moreover, research also suggests that ADHD is also associated with changes in how the cerebellum interacts with other brain regions, including the cerebral cortex (Qiu et al., 2011). This may in turn explain some of the changes in cognition seen in cerebellar patients.

In summary, abnormal cerebellar development, morphological differences of the cerebellum and cerebellar connectivity, and results from behavioral studies where the cerebellum is involved prove that this structure of the brain is clearly involved with ADHD.

#### Neurocognitive functions in ADHD

In the following section, we analyze studies that examine the neurocognitive functions of patients with ADHD. The research indicates that patients with ADHD experience deficits across all cognitive domains, although the specific subdomains affected differ from those observed in the other disorders reviewed.

#### Executive functions

Studies have shown differences in neurocognitive function between patients with ADHD and controls, as evidenced by results from the 3-dimensional computerized Tower of London Test. This test measures both planning latencies and accuracy and has demonstrated that patients with ADHD exhibit impairments in both areas. This trend of impairment is thought to stem from difficulties in inhibiting responses when faced with problem-solving tasks, resulting in decreased planning activity (Young et al., 2007). The working memory capacity, which is also important for planning ability of patients with ADHD was evaluated using the Tower of London-Freiburg Version, and the results showed no significant differences between the ADHD patients and healthy controls (Mohamed et al., 2021). The Stroop Test, which measures patient’s ability to shift attention and inhibit interfering information (MacLeod, 1991), was conducted to assess the inhibitory ability of ADHD patients.

Results showed that patients with ADHD exhibit impaired inhibition compared to healthy controls, as reported in various studies (Fabio and Caprì, 2017; Guo et al., 2021; Callahan et al., 2022). However, it should also be noted that some studies have found no differences in executive control and inhibitory ability between ADHD patients and healthy controls, as indicated by similar error rates (Aycicegi-Dinn et al., 2011). While some tests suggest impairments in cognitive flexibility, attentional switching, and working memory, others find no significant differences between ADHD patients and healthy controls. For example, the Trail Making Test-Langensteinbacher Version showed no impairments in cognitive flexibility and attentional switching in ADHD patients (Mohamed et al., 2021). In contrast, adults with ADHD performed worse on Trial Making Test Part B used to measure visual attention and task switching (Tatar and Cansız, 2022). Cognitive flexibility and reaction time were measured with the Cognitive Flexibility Inventory and the Iowa Gambling Task, results were significantly lower in the ADHD patients compared to the control group (Roshani et al., 2020). Studies evaluating working memory using various tests, including the 2-Back Test, the Vienna Test System (Schellig and Schuri, 2009), and the Tower of Hanoi Test (Fabio and Caprì, 2017), also produced contradictory results. While some studies found impairments in working memory function in ADHD patients (Fabio and Caprì, 2017; Nikolas et al., 2019), others found no significant differences (Mohamed et al., 2021). The Letter/Number Sequencing Task (Gold et al., 1997) was used to assess auditory working memory, and results showed that ADHD patients had significantly lower scores compared to healthy controls (Aycicegi-Dinn et al., 2011). In contrast, visuospatial working memory, which does not require auditory processing, was found to be similar between ADHD patients and controls (Aycicegi-Dinn et al., 2011). Studies have linked deficits in executive function in ADHD patients specifically to the cerebellum. In an fMRI study, Depue et al. (2010) showed that ADHD patients with executive dysfunction had reduced cerebellar activation and decreased cerebellar volume, plus decreased activation of the frontal cortex and basal ganglia compared to healthy controls.

Another fMRI study by Hirose et al. (2014), showed changes in cerebro-cerebellar interaction during response inhibition in task performances. This suggests that the involvement of the cerebellum in the functional enhancement of inhibitory control may be sustained by modifications of the prefrontal-cerebellar interactions.

#### Attention and processing speed

Patients with ADHD exhibit difficulties with processing speed, distractibility, and selective attention, leading to an increased number of omission errors compared to healthy controls on tests such as the Vigilance and Sustained Attention Test and Selective Attention Test, two subtests of the Vienna Test System (Sturm, 2017; Mohamed et al., 2021). Similarly, ADHD patients showed a clear impairment in the Continuous performance test which is used to measure sustained attention and response inhibition (Tatar and Cansız, 2022). Performance deficits in patients with ADHD were seen for errors of omission, correct responses, late responses, and errors of commission compared to healthy controls. Low scores emerged using the non-conflict block of the Go/No-Go task and the Stroop Color-Word Task to assess processing speed. Taken together these results have shown that ADHD patients have slower response times compared to healthy controls (Aycicegi-Dinn et al., 2011). Furthermore, research has consistently demonstrated that sustained attention, reaction time, inattention, impulsivity, response speed and variability of reaction time are significantly impaired in patients with ADHD (Tinius, 2003; Nikolas et al., 2019; Guo et al., 2021). Like with executive functions, deficits in attention and processing speed have been linked to the cerebellum. For example, structural neuroimaging studies have shown reduced cerebellar volumes in ADHD which in turn correlates with attentional problem (Petacchi et al., 2005; Kucyi et al., 2015).

#### Language

The results of verbal fluency tests in patients with ADHD are inconsistent. While some studies have found no difference between healthy controls and ADHD patients using the Resensburg Word Fluency Test (Aycicegi-Dinn et al., 2011; Mohamed et al., 2021), others have reported impaired verbal fluency when different testing methods were used (Hervey et al., 2004; Boonstra et al., 2005; Tucha et al., 2005; Guo et al., 2021). The naming ability of patients with ADHD, which is related to semantic memory retrieval capacity, has not yet been fully explored in terms of its contribution to language in this neuropsychiatric disorder.

Children with ADHD showed slower speed response than typically developed children on a naming test (Wang et al., 2017a). Furthermore, ADHD children show lower accuracy, higher number of errors and slower responses in figure naming compared to healthy controls (Koltermann et al., 2020). A previous study by Takeda et al. (2020) attempted to identify differences in Wechsler Adult Intelligence Scale profiles between ADHD patients and healthy controls, but no significant group differences were found in Verbal intelligence quotient. However, a more recent study by Doi et al. (2022) found that female ADHD patients had a lower Verbal intelligence quotient compared to males when characterizing the cognitive profile of ADHD and ASD.

The contribution of the cerebellum, together with other brain areas are necessary for language processing. Verbal fluency and lexical retrieval tasks are associated with cerebellar activation. For example, the contralateral cerebellar hemisphere, together with Broca’s area, was actively involved in the production of semantically related verbs in response to visually presented nouns. This is an example how these actions are due to non-motor cognitive processes subserving semantic word association (Petersen et al., 1988, 1989; Stoodley and Schmahmann, 2009a; De Smet et al., 2013). Further reinforcing the cerebellums role in language, Arasanz et al. (2012) demonstrated that continuous cerebellar theta burst stimulation (cTBS) using Transcranial Magnetic Stimulation (TMS) interfered with performance on phonemic and semantic fluency tasks.

#### Memory

ADHD patients have shown lower scores in delayed recall of verbal memory compared to healthy controls as tested by the Verbal Learning and Memory Test (Mohamed et al., 2021). Likewise, immediate and delayed verbal recall, tested using the Logical Memory Subtest of the Wechsler Memory Scale, have been found to be clearly impaired in ADHD patients (Aycicegi-Dinn et al., 2011; Callahan et al., 2022). Further, the immediate and delayed visual recall tested using the Rey-Osterrieth Complex Figure test showed that ADHD patients performed worse than healthy controls (Aycicegi-Dinn et al., 2011). On the other hand, Dinn and colleagues found that patients with ADHD do not have a memory loss once the information is encoded (Aycicegi-Dinn et al., 2011).

A meta-analysis investigating the presence of procedural sequence learning found that ADHD patients do not exhibit significant deficits and that procedural sequence learning appears to be intact in ADHD (Sanjeevan et al., 2020). However, another study reported impaired results for procedural memory (Butzbach et al., 2019).

The cerebellum also contributes to memory functions, for example, Fastenrath et al. (2022) show that primarily the amygdala and successively the cerebellum is part of a network implicate in emotional enhancement of episodic memory and as well the right cerebellum is involved in semantic memory task (Gatti et al., 2021).

#### Visuospatial perception and visuospatial ability

Visuospatial ability, tested with the Rey-Osterrieth Complex Figure test, did not show significant differences between healthy controls and ADHD patients (Aycicegi-Dinn et al., 2011; Callahan et al., 2022). Schreiber et al. (1999) suggested that lower results on Rey-Osterrieth Complex Figure test copy trial was due to executive dysfunction in adults with ADHD and Rey-Osterrieth Complex Figure test can be a sensitive test to detect these deficits.

#### Social cognition

Social cognition is still poorly explored in patients with ADHD, in particular regarding adults (Morellini et al., 2022). Contrasting results in the literature emerge for this cognitive domain. Some studies demonstrated that emotion recognition and processing, emotional prosody and empathy are impaired in ADHD patients. Control groups performed better than ADHD patients on the Cambridge Behavior Scale, the Tübingen Affect Battery and Reading mind from the eyes test (Uekermann et al., 2010; Kis et al., 2017; Tatar and Cansız, 2022). In contrast, healthy controls and ADHD patients did not differ on theory of mind task like the revised version of Reading the Mind in the Eyes Test and the Object Alternation Test (Aycicegi-Dinn et al., 2011).

Cerebellum plays a fundamental role in identifying sequences in movements that are important to understand the intentions of others. For example, mirroring, attribute mental states in others, mentalizing, and making predictions regarding social behavior (Van Overwalle et al., 2020). Interestingly, the same functions are impaired in patients with ASD diagnosis (see below).

#### General intelligence

Intelligence is not impaired in patients with ADHD. Researchers who used the Cattell’s Culture Fair Intelligence Test found no difference between healthy controls and adult ADHD patients (Aycicegi-Dinn et al., 2011). Likewise, Takeda et al. (2020) showed that intelligence quotient was not impaired in adults with ADHD, although working memory and processing speed indices of Wechsler Memory Scale-III were significantly lower than healthy controls.

#### Summary

Adults with ADHD exhibit clear impairments in multiple cognitive domains that impact their daily functioning. The cognitive heterogeneity among adults with ADHD may be due to variations in comorbid disorders, medication status, and mood disorders within the ADHD group (Cumyn et al., 2009; Chen et al., 2018).

An overview of the neurocognitive functions and how they are affected in ADHD according to this review are illustrated in Table 1. Executive functions, such as the ability to inhibit, cognitive flexibility, as well as the processing of auditory information, are particularly compromised in these patients. On the other hand, visuospatial working memory is less impacted than auditory working memory as it does not require additional auditory processing (Aycicegi-Dinn et al., 2011).

Several deficits were identified under the attention cognitive domain and processing speed as well ias verbal fluency tasks (Hervey et al., 2004; Boonstra et al., 2005; Tucha et al., 2005; Guo et al., 2021). Visual episodic memory appears to be less affected compared to verbal episodic memory in patients with ADHD. However, impairments in verbal delayed recall and visual immediate and delayed recall have been observed, but recognition ability appears to be preserved irrespective of modality (Aycicegi-Dinn et al., 2011; Callahan et al., 2017; Butzbach et al., 2019; Sanjeevan et al., 2020; Mohamed et al., 2021). Hence, the ability to store (transfer?) information to the declarative long-term memory is not affected, but rather the retrieval of information.

The results of social cognition studies in patients with ADHD are contradictory, and the presence of undiagnosed comorbid ASD could be a confounding factor that negatively impacts their social cognition abilities (Uekermann et al., 2010; Aycicegi-Dinn et al., 2011; Morellini et al., 2022).

Mental rigidity, a common feature in patients with ADHD and ASD, could also be contributing to the varying results. The presence of disinhibition, difficulties with planning, and lack of motivation, as well as other dysexecutive symptoms characteristic of frontal lobe syndrome, have been reported among patients diagnosed with ADHD. These symptoms can impact other cognitive domains, and self-report questionnaires have shown high scores for executive dysfunction, indicating deficits in neuropsychological assessments (Aycicegi-Dinn et al., 2011).

When it comes to ADHD, the presence of undiagnosed comorbidities and other psychiatric disorders can significantly impact the results of neurocognitive assessments. Additionally, the consumption of psychotropic substances and multiple medications by some ADHD patients can greatly impact their cognitive and executive function performance (Vik et al., 2004). For instance, in a study done by Crunelle et al. (2013), ADHD patients that used cocaine had higher levels of motor and cognitive impulsivity compared to healthy controls.

Collectively, the evidence from behavioral and neuroimaging studies, indicates that ADHD is associated with cerebellar abnormalities and that these cerebellar abnormalities impact cognitive functions. This suggests that the symptoms associated with ADHD are partly due to changes in cerebellar function. However, it is also clear that ADHD is a disorder that affects multiple brain regions, and more research is required to determine the precise nature of the relationship between these deficits and the extent to which they are due to cerebellar dysfunction or problems with the interplay between various brain regions, including the cerebellum. Identifying the specific contribution of cerebellar impairment in the context of ADHD is crucial to advance our understanding of this disorder and inform the development of effective interventions to improve cognitive and motor functions.

### Autism spectrum disorder

Autism spectrum disorder is characterized by difficulties with social interaction, communication, limited interests, and repetitive and restrictive behavior (Hodges et al., 2020). These problems can be of varying degrees and be associated with disorders of language development, general intelligence, emotional regulation, sensory sensitivity, and executive abilities. Sometimes intelligence and general cognitive functioning are preserved (Robertson and Baron-Cohen, 2017; Cai et al., 2018; Demetriou et al., 2018). There is no pharmacological treatment for ASD. However, early diagnosis and intervention coupled with remedial services can benefit patients (Baio et al., 2018).

The most common suggested causes of ASD are metabolic and physiological disorders, immunological dysfunction, oxidative stress, and mitochondrial dysfunction (Nardone and Elliott, 2016). Altered sensory processing and perception have been one of the characteristics of ASD (Cheung and Lau, 2020). Patients with ASD are extra sensitive to the sensory input of all modalities. This sensitivity reflects differences in sensory and cognitive processing as well as differences in low-level processing in sensory-devoted areas (Robertson and Baron-Cohen, 2017; Cai et al., 2018).

Autism spectrum disorder has many comorbidities, including morphological abnormalities like macrocephaly, cerebral palsy, physiological impairments like gastrointestinal, sleep problems, psychiatric disorders like anxiety, ADHD, obsessive-compulsive disorder, tic disorders, Tourette syndrome, epilepsy, and Rett syndrome (Sharma et al., 2018; Al-Beltagi, 2021). Patients who have shown delayed development, such as late language development, lack of interest in social interaction with other children, weak motor ability, and abnormal eye contact, have an increased risk of having ASD (Miniscalco et al., 2006). In severe cases, the child does not develop normal language, lacks social interaction, or has deviant behavior such as repetitive movements or compulsive attachment to particular objects. Patients with ASD show stereotypes, rituals, and mental rigidity (Esbensen et al., 2009). Occasionally hallucinations, delinquency and self-harming behavior emerge (Lai et al., 2014). Patients with ASD and normal language development can go undetected into adulthood (Gilchrist et al., 2001). Patients with ASD also often have social and emotional problems and may exhibit intense periods of motor anxiety and irritability typically triggered by changes in routines and the environment or sensory overload (Yuan et al., 2022). For them, intuitive thinking is often altered, and they often have great difficulties reading body language, understanding irony, and interpreting non-verbal signals (Noens and van Berckelaer-Onnes, 2005).

Executive dysfunction can contribute to the symptoms associated with ASD (Demetriou et al., 2018; Jones et al., 2018). The prevalence of autistic traits is normally distributed in the population. For a diagnosis, the symptoms and problems must have been consistent since childhood, be clear in all or most contexts, and lead to a significant disability in several functional domains (Cai et al., 2018; Styles et al., 2020).

Oristaglio et al. (2013) tested eyeblink conditioning on children with ASD, and they showed abnormally timed conditioned blink responses compared to the control group. Different diagnostic subgroups of ASD like Asperger’s syndrome, pervasive developmental disorder, and typical ASD, displayed differences in the timing of conditioned responses during trace and delay eyeblink conditioning (Oristaglio et al., 2013; Welsh and Oristaglio, 2016). Differences have also been observed in finger tapping–another task that has been linked to cerebellar functioning. Compared to controls, patients with ASD showed altered motor performance and temporal processing instability (Morimoto et al., 2018). In line with the cerebellum importance of finger tapping, children with ASD showed a reduced activation of the ipsilateral cerebellum while performing the task (Mostofsky et al., 2009).

#### Cerebellum and ASD

Post-mortem and structural MRI studies have highlighted that the frontal lobes, amygdala and cerebellum are abnormal in ASD (Amaral et al., 2008). Structural brain differences have been reported in terms of increased total brain volume, especially in young children with ASD, and regional gray/white matter differences in both adults and children with ASD. However, the results are not totally clear because of variations in diagnostic set-up, inclusion criteria, age, and IQ of participants. Voxel-based morphometry has revealed gray matter increases in brain areas implicated in social cognition, communication, and stereotyped behaviors–the core atypical features of ASD (Hyde et al., 2010). A number of changes has also been reported in the cerebellum in patients with ASD, including abnormal development of the cortex, reduced numbers of Purkinje cells, and hypoplasia without cogent gliosis (Bauman and Kemper, 2003; Whitney et al., 2008). These changes may in part be caused by an increment of interleukin 6 that acts as both a pro-inflammatory cytokine and an anti-inflammatory myokine and causes functional deficits of cerebellar circuits (Wei et al., 2011). Other studies support the hypothesis that a reduced release of dopamine in the frontal cortex is due to alteration in the cerebellum and with a decrease in the functional connectivity between the cerebellum and the cerebral cortex (Mittleman et al., 2008).

In addition to these morphological differences, there are many studies showing that the performance of patients with ASD differs from controls on tasks related to the cerebellum. For example, performance on eyeblink conditioning, a simple form of learning that is dependent on the cerebellum, differs between patients with ASD and controls (Sears et al., 1994; Welsh and Oristaglio, 2016). Eye-tracking to assess cerebellar integrity and assessment of an eventual comorbid epilepsy may allow for better sub-phenotyping within the heterogeneous populations that comprise ASD (Bozzi et al., 2018; Freedman and Foxe, 2018). Freedman and Foxe (2018) reported in their review a reduced size of the cerebellar vermis in ASD, which could critically impact visuo-sensorimotor development in early ages, and disrupt visual orienting, communication, and social interaction. ASD patients showed lower saccades movement and less peak speeds than controls on volitional saccade task (Stanley-Cary et al., 2011). Differences in velocity and shift in saccade dynamics typically occurs during adaptation in ASD participants (Johnson et al., 2013). Behavioral studies on mice with distinct degrees of developmental Purkinje cell loss show a correlation between the number of Purkinje cells and the degree of behavioral rigidity on learning tasks designed to assess autistic behavior in mice (Dickson et al., 2010; Martin et al., 2010). Damage to the cerebellum at the beginning of development can have long-term effects on movement, cognition, and emotional regulation. These effects may in part reflect disruption of interaction between the cerebellum and other brain regions (Wang et al., 2014). Abnormalities in different cerebellar subregions produce specific behavioral symptoms related to the functional disruption of specific cerebro-cerebellar circuits and influence brain structural development (Stoodley, 2014, 2016).

Taken together, ample evidence shows cerebellar impairment in patients and mouse models with ASD. Cerebellar abnormalities cause disruption of functions related to the cerebellum, but they may also negatively affect the ability to interpret external stimuli and organize internal processes. This doesn’t mean that the fundamental cause of ASD is to be found within the cerebellum, but rather that whatever neural alterations lead to ASD, the cerebellum likely plays an important role.

### Neurocognitive functions in ASD

#### Executive functions

Rumsey (1985) started to explore executive functions deficits in ASD. He demonstrated that patients with a clear ASD diagnosis compared to healthy controls displayed an increased number of perseverative responses on the Wisconsin Card Sorting Test, used to measure abstract reasoning capacity and the ability to shift cognitive strategies. Perseverative responses can be due, partially to difficulties with response inhibition and to cognitive flexibility, which can be observed in patients with ASD in everyday resistance to change, and difficulty with shifts between activities. Russo et al. (2007) summed up and analyzed several studies in order to clarify inhibition’s ability, working memory and cognitive flexibility in ASD patients; results concerning these functions were variable but, however, impaired. ASD patients also have significant deficits with control of interference, motor timing and ability to inhibit an automatic response (Hlavatá et al., 2018). In another study motor inhibition ability was also impaired (Hughes et al., 1994).

Neuropsychological studies demonstrated deficits in working memory and other executive dysfunctions for patients affected by ASD compared to healthy controls (Hill, 2004; Kercood et al., 2014; Marinopoulou et al., 2016). Results of neuropsychological assessments were significantly lower concerning cognitive flexibility, planning ability, auditory working memory and visuospatial working memory in ASD patients compared to controls (Geurts et al., 2004; Goldberg et al., 2005; Landa and Goldberg, 2005). Wilson et al. (2014) used the Non-word Repetition task to assess phonological working memory, speech perception, phonological assembly, and short-term memory. Test results did not show significant differences between healthy controls and patients with ASD.

There is growing evidence that the changes in executive functions observed in ASD patients are linked to changes in the connectivity between the cerebellum and the cerebrum (Gilbert et al., 2008). For example, Bednarz and Kana (2019) highlighted cerebellar contribution to altered network connectivity in ASD. Intrinsic connectivity networks such as the executive control network, the default mode network, and salience networks are particularly important for cognitive processing.

#### Attention and processing speed

Patients with ASD scored significantly lower than healthy controls on the Purdue Pegboard Test, a psychomotor test of manual dexterity and bimanual coordination also used to measure executive functions and attention (Wilson et al., 2014). ASD patients demonstrate clear impairments compared to healthy controls for attentional set-shifting and planning measured, respectively, with Intra-Dimensional Extra-Dimensional set shifting task and Tower of London test (Hughes et al., 1994). Adults with ASD have been shown to have evident deficits in the processing speed subtests of the WAIS-III and WAIS-IV (Marinopoulou et al., 2016; Braconnier and Siper, 2021). In contrast, previous studies did not find any impairment in this cognitive domain (Minshew et al., 1997).

#### Language

Evident abnormalities were previously identified in the domain of the verbal functions. Difficulties with social interaction, verbal and non-verbal communication, and imaginative activities consistently occurred together with language difficulties (Wing and Gould, 1979). Adults with ASD have been shown to have deficits in verbal reasoning subtests of the WAIS-IV, the total Verbal Intelligence Quotient of ASD patients were found to be much lower than other cognitive quotients and this could be used as a possible ASD predictor (Braconnier and Siper, 2021; Johnson et al., 2021). Previous clinical research showed no impairment in verbal fluency that was tested with the FAS task on ASD patients (Wilson et al., 2014). The use of complex language and reasoning domains showed impairments in ASD patients (Minshew et al., 1997). The naming ability of patients with ASD, which is related to semantic memory retrieval capacity, has not yet been fully explored in terms of its contribution to language. Recent results in a neuropsychological study on children with ASD demonstrate that their results did not differ from healthy controls on naming accuracy and naming speed (Fletcher et al., 2020).

#### Memory

Williams et al. (2017) tested memory functions on children and adults with ASD using the Logical Memory subtest of the Wechsler Memory Scale-III and the Wide Range Assessment of Memory and Learning. Their study results for adults with ASD showed that on the WMS recall score for story details there was a significant main effect for immediate, short-term delay and long-term delay conditions. For adult thematic scores there was a relatively small discrepancy between the ASD and healthy controls on immediate recall but there was a clear discrepancy on long-term memory. Results for children and adolescent with ASD indicated that they did not differ from healthy controls in the percentages of patients with relative impairment at the immediate or 30-min delay intervals; but the ASD group had a significantly higher percentage of patients with relative impairment at the 2-day delay interval (Williams et al., 2017). Decreased story themes reflect the use of underlying organizational strategies.

In a recent study Griffin et al. (2022) focused on evaluating episodic memory in ASD; they showed that immediate recall, delayed recall and recognition are impaired in both auditory and visual modalities. General deficits were identified in the prospective memory in ASD, performance was impaired in time-based tasks and event-based cues of non-focality or low salience, that require high cognitive functioning and elevated attention (Sheppard et al., 2018).

#### Visuospatial perception and visuospatial ability

In a previous study there were no significant performance differences between healthy controls and ASD patients on the Embedded Figures Test that measure local/global perceptual style, central coherence and perception ability (Wilson et al., 2014). According to previous studies visuospatial abilities seem not be impaired (Minshew et al., 1997). Adults with ASD have been shown to have deficits with the visuospatial perception task of the WAIS-IV (Braconnier and Siper, 2021). Visual detection, motion perception, and face processing are altered in ASD, where attention deficits and social brain network impairment influence these three brain functions (Chung and Son, 2020). Guy et al. (2019) studied visuospatial perception on patients with ASD using the Navon task. The results did not differ for reaction times between controls and ASD patients, but the effect of interference was significantly higher in participants with ASD. Other clinical researches which were focused to clarify visuoperceptual functions did not demonstrate any impairment but instead that patients with ASD have an enhanced ability to process local or detailed stimuli (Mottron and Burack, 2001; Behrmann et al., 2006; Mottron et al., 2006).

#### Social cognition

Social cognition seems to be one of the most impaired cognitive domains for patients affected by ASD. Many studies demonstrated a strong impairment with social cognition in ASD patients, where, respectively, the theory of mind, emotion processing and social orientation were deficient (Happé et al., 2006; Enticott et al., 2014; Wilson et al., 2014; Griffiths et al., 2019). Patients with ASD performed more poorly than healthy controls on Frith-Happé Animations Test, on Karolinska Directed Emotional Faces test and on “Reading the Mind in the Eyes” Task used for emotion recognition (Wilson et al., 2014). Social skill and social motivation are robustly impaired in ASD patients compared to non-ASD controls (Morrison et al., 2020). In a previous study the Awareness of Social Inference Test Part 2A has been used to assess explicit theory of mind and social perception; results showed that patients with ASD had deficits with regard to complex social perception compared to their matched controls (Veddum et al., 2019). Executive dysfunctions also negatively influence social cognition in ASD (Pellicano, 2007; Freeman et al., 2017). Low cognitive abilities and in particular executive dysfunctions affect adequate responses to the environment and the ability to learn from social interactions (Sarovic, 2021).

#### General intelligence

Social, cognitive, and adaptive skill deficits characterize intellectual disabilities, which are present in this neuropsychiatric and neurodevelopmental disorder (Downs et al., 2008; Matson and Shoemaker, 2009). The normal population shows higher cognitive ability in terms of intelligence level and executive functions (La Malfa et al., 2004; Russo et al., 2007; Wang et al., 2017b). Around 45% to 75% of patients with ASD have an intellectual disability and from 20% to 40% of patients with intellectual disability turn out to have ASD (La Malfa et al., 2004; Saemundsen et al., 2010; Lai et al., 2014). Patients with ASD have their cognitive strengths and weaknesses as well as variable intelligence levels. Dawson et al. (2007) tested ASD children with the Raven’s Progressive Matrices to measure crystallized intelligence, performances on this test were spread on the lower and higher parts of the average from 30th percentiles to 70th percentiles (Dawson et al., 2007). Within the entire ASD, there are patients with a low intelligence quotient and also over-talented patients. However, the average intelligence quotient is significantly lower than in the general population, probably around 80 rather than 100. The diagnosis of ASD is generally associated with an intellectual quotient below 80 (Gillberg and Rasmussen, 2011). Thus, it could be more appropriate to distinguish ASD patients with cognitive and intellectual disability and ASD patients with a normative or above average IQ.

#### Summary

The neurocognitive functions in ASD are summarized in Table 1. Multiple cognitive functions in ASD are impaired like abstract reasoning capacity, cognitive flexibility, inhibition ability, working memory, planning ability, attention, verbal functions, process speed, emotion processing, social cognition, immediate recall, delayed recall, and recognition in both visual and verbal modalities (Hughes et al., 1994; Geurts et al., 2004; Hill, 2004; Goldberg et al., 2005; Russo et al., 2007; Wilson et al., 2014; Hlavatá et al., 2018; Sheppard et al., 2018; Griffin et al., 2022). Executive dysfunctions significantly decrease the life quality of patients affected by ASD (de Vries and Geurts, 2015). A major challenge when studying ASD is that ASD is a heterogenic developmental disorder, resulting in high variability on cognitive tests (Newschaffer et al., 2002). ASD often co-occurs with ADHD and patients with both diagnoses often have a different pattern of symptoms (Leitner, 2014; Ashwood et al., 2015). Other medical conditions like hyperkinetic disorders, obsessive-compulsive disorder, Tourette’s syndrome, language delay, epilepsy, depression, anxiety, schizophrenia, bipolar disorder, personality disorder, fragile X syndrome, tuberous sclerosis, or general learning disability can be critical confounders that affect performance on neuropsychological assessments. Attention deficits often cause fatigue and reduce the patient’s cognitive performance (Zwick, 2017). Repetitive behavior and rigid mentality have been associated with caudate abnormality in ASD (Sears et al., 1999). The neurocognitive profile of adults with ASD is, however, still partially unclear. As for ADHD, evidence suggests that ASD is associated with changes in multiple brain regions, including the cerebellum. Combined with studies showing that ASD is associated with deficits in tasks related to cerebellar function, there is convincing evidence that the symptoms associated with ASD are partly due to cerebellar abnormalities. Again, future studies need to address the extent to which these deficits are linked directly to the cerebellum versus the interplay between the cerebellum and other parts of the brain.

## Discussion

This review highlights differences in neurocognitive functions, illustrated in Table 1, of two disorders and one disease affecting the cerebellum – ADHD, ASD, and SCA3. Multiple cognitive impairments are evident in all three conditions (Barnard et al., 2008; Guo et al., 2021; Yap et al., 2022b). The cognitive impairments associated can exacerbate functional deficits, leading to significant negative impacts on daily life, socioeconomic status, and personal relationships.

These three conditions are all associated with clear cerebellar abnormalities, which may in turn affect cognitive functions, directly or indirectly. Although progress has been made in identifying cognitive difficulties, further research is needed to fully understand the disorders and their effects on other cognitive functions. Currently, many of the cognitive difficulties are not sufficiently acknowledged, resulting in limited recognition and understanding in society. A key goal of future research should be to determine if these cognitive deficits are directly caused by the disorders, by other comorbid conditions, or a combination of both.

Other conditions such as low or high intelligence quotient, epilepsy, tics, depression, anxiety, eating disorders, obsessive-compulsive disorder, and burnout syndrome can impact the performance of patients with SCA3, ADHD, and ASD on neuropsychological assessments. The presence of these conditions can be confounding factors that clinicians must take into account when interpreting test results and associated cognitive functions. Other problems with earlier studies include small sample sizes, poor neurocognitive task selection, cultural differences and patient heterogeneity; which may also contribute to the variations in performances revealed in this review.

There are several reasons why it is important to assess neurocognitive function in ADHD, ASD, and SCA3. First, identifying cognitive deficits that arise during the early stages of these conditions could help prevent or alleviate further cognitive impairments. Second, understanding the cognitive challenges facing these patients can help caregivers as well as the patients themselves to facilitate their daily routines. Third, the identification of cognitive deficits can be a starting point for personalized work and educational rehabilitation programs. Failure to detect cognitive impairments, on the other hand, can have severe effects. Cognitive deficits can trigger high levels of psychosocial stress at school or work which can lead to depression, anxiety, and ultimately self-harm, violence, and increased risk of suicide.

Neuropsychological assessments can contribute to the discrimination between many disorders and diseases, for example, Alzheimer’s disease, frontotemporal dementia, and post-traumatic stress syndrome. However, for ADHD, SCA3, and ASD there are few neuropsychological assessments used in clinical practice. The neurocognitive functions have not been extensively evaluated and clarified in SCA3. Future studies need to clarify how the damage to the cerebellum affects cognitive functions with the support of neuroimaging, how cerebellar damage in SCA3 affects the neurocognitive domains, and if it is possible to detect a mild cognitive impairment which often leads to cognitive diseases or dementia (Teive and Arruda, 2009; Lindsay and Storey, 2017).

For ADHD the Integrated Visual and Auditory continuous performance test is commonly used by researchers and clinicians (Tinius, 2003; Arble et al., 2014). This test has good specificity and sensitivity for categorizing ADHD type. However, a disadvantage of this continuous performance test is its relatively long duration of 15 min without interruption, resulting in many patients not completing the test. Therefore, other tests are needed in clinical evaluations that can measure the same functions but without these disadvantages. Neuroimaging has shown promise as a quantitative and non-invasive method for the assessment of structural and functional brain alterations in clinical SCA3 patients as well as preclinical carriers. Neuropsychological assessments can as well contribute to identifying a possible hidden mild cognitive impairment, when there are no clear biomarkers but light cognitive symptoms that clinicians often miss (Stezin et al., 2021). Furthermore, neuropsychological assessments can contribute to giving a comprehensive diagnostic, functional description, and evaluate the severity of the symptoms, supported by cognitive profiles.

However, for neuropsychiatric disorders like ADHD and ASD neuroimaging results have been less clear, perhaps because of the heterogeneity of these disorders (Anagnostou and Taylor, 2011; Cortese and Castellanos, 2012; Wolff et al., 2018; Giedd, 2019; Pereira-Sanchez and Castellanos, 2021). ADHD and ASD are diagnosed on the basis of behavioral symptoms and clinical history but cognitive abilities may also be useful in characterizing patients with ADHD and ASD.

## Future directions

Future studies are needed to further explain the role of the cerebellum in both normal and pathological behavior, mood regulation, impulse control and how it contributes to executive cognitive functioning. Further, new clinical studies investigating the cognitive profiles of ADHD, ASD, and SCA3, are necessary to gain insight into the nuances of the disorders which in turn can improve the differential diagnosis and the potential to identify sub-cognitive phenotypes of patients with ADHD and ASD, which in turn may help us develop better treatments and strategies. Non-pharmacological strategies could also help the large group of patients with neuropsychiatric symptoms who do not fulfill all the diagnostics criteria to obtain a formal ADHD or ASD diagnosis. Given that most of the studies on ASD have focused on children or adolescents, further studies are needed to clarify the cognitive functions of adults with ADHD and ASD.

To fully understand how the cerebellum works we must study how it contributes to different psychological functions and neurocognitive domains. Considering the rich cerebro-cerebellar connections, we should expect that changes in the cerebellum can affect cognitive functions such as verbal functions, cognitive flexibility, impulse control, task initiation, efficiency, self-monitoring, planning, and prioritizing. The findings from neuroimaging and neuropsychology reviewed here show cogent evidence that the human cerebellum plays a pivotal role in many cognitive functions. Accordingly, ADHD, ASD, and SCA3–three conditions associated with abnormalities in cerebellar morphology–all affect various cognitive functions, although more research is needed to uncover the causal relationships involved. Future clinical studies on these patient groups and other patients in which the cerebellum is affected can help us uncover the cerebellum’s role in cognition and lead to an improved understanding of the difficulties facing patients with cerebellar alterations.

## Author contributions

MC: wrote the first draft, conception, disposition, and organization of the work. SV: reviewed and critiqued the parts inherent to cognition, neurocognitive impairments, and neuropsychology. PG: reviewed and critiqued the parts inherent to ADHD and ASD. SG: reviewed and critiqued the parts inherent to ataxia and SCA3. AR: wrote the final draft, supervised the main work, conception, financing, and organization of the work. All authors contributed to the article and approved the submitted version.

## References

[B1] Adamaszek M. D’Agata F. Ferrucci R. Habas C. Keulen S. Kirkby K. C. (2017). Consensus paper: Cerebellum and emotion. *Cerebellum* 16 552–576. 10.1007/s12311-016-0815-8 27485952

[B2] Ahmadian N. van Baarsen K. van Zandvoort M. Robe P. A. (2019). The cerebellar cognitive affective syndrome-a meta-analysis. *Cerebellum* 18 941–950. 10.1007/s12311-019-01060-2 31392563 PMC6761084

[B3] Al-Beltagi M. (2021). Autism medical comorbidities. *World J. Clin. Pediatr.* 10 15–28. 10.5409/wjcp.v10.i3.15 33972922 PMC8085719

[B4] Alexander G. E. DeLong M. R. Strick P. L. (1986). Parallel organization of functionally segregated circuits linking basal ganglia and cortex. *Annu. Rev. Neurosci.* 9 357–381. 10.1146/annurev.ne.09.030186.002041 3085570

[B5] Allen G. I. Tsukahara N. (1974). Cerebrocerebellar communication systems. *Physiol. Rev.* 54 957–1006. 10.1152/physrev.1974.54.4.957 4370744

[B6] Amaral D. G. Schumann C. M. Nordahl C. W. (2008). Neuroanatomy of autism. *Trends Neurosci.* 31 137–145. 10.1016/j.tins.2007.12.005 18258309

[B7] Anagnostou E. Taylor M. J. (2011). Review of neuroimaging in autism spectrum disorders: what have we learned and where we go from here. *Mol. Autism* 2:4. 10.1186/2040-2392-2-4 21501488 PMC3102613

[B8] Anbarasan D. Kitchin M. Adler L. A. (2020). Screening for Adult ADHD. *Curr. Psychiatry Rep.* 22:72. 10.1007/s11920-020-01194-9 33095375

[B9] Ankri L. Husson Z. Pietrajtis K. Proville R. Léna C. Yarom Y. (2015). A novel inhibitory nucleo-cortical circuit controls cerebellar Golgi cell activity. *eLife* 4:e06262. 10.7554/eLife.06262 25965178 PMC4461794

[B10] Apps R. Garwicz M. (2005). Anatomical and physiological foundations of cerebellar information processing. *Nat. Rev. Neurosci.* 6 297–311. 10.1038/nrn1646 15803161

[B11] Arasanz C. P. Staines W. R. Roy E. A. Schweizer T. A. (2012). The cerebellum and its role in word generation: a cTBS study. *Cortex* 48 718–724. 10.1016/j.cortex.2011.02.021 21457953

[B12] Arble E. Kuentzel J. Barnett D. (2014). Convergent validity of the Integrated Visual and Auditory Continuous Performance Test (IVA+Plus): associations with working memory, processing speed, and behavioral ratings. *Arch. Clin. Neuropsychol.* 29 300–312. 10.1093/arclin/acu006 24687587

[B13] Argyropoulos G. P. D. van Dun K. Adamaszek M. Leggio M. Manto M. Masciullo M. (2020). The cerebellar cognitive affective/schmahmann syndrome: A task force paper. *Cerebellum* 19 102–125. 10.1007/s12311-019-01068-8 31522332 PMC6978293

[B14] Arruda W. O. Meira A. T. Ono S. E. de Carvalho Neto A. Betting L. E. G. G. Raskin S. (2020). Volumetric MRI Changes in Spinocerebellar Ataxia (SCA3 and SCA10) Patients. *Cerebellum* 19 536–543. 10.1007/s12311-020-01137-3 32367276

[B15] Ashida R. Cerminara N. L. Edwards R. J. Apps R. Brooks J. C. W. (2019). Sensorimotor, language, and working memory representation within the human cerebellum. *Hum. Brain Mapp.* 40 4732–4747. 10.1002/hbm.24733 31361075 PMC6865458

[B16] Ashizawa T. Xia G. (2016). Ataxia. *Continuum* 22 1208–1226. 10.1212/CON.0000000000000362 27495205 PMC5567218

[B17] Ashtari M. Kumra S. Bhaskar S. L. Clarke T. Thaden E. Cervellione K. L. (2005). Attention-deficit/hyperactivity disorder: a preliminary diffusion tensor imaging study. *Biol. Psychiatry* 57 448–455. 10.1016/j.biopsych.2004.11.047 15737658

[B18] Ashwood K. L. Tye C. Azadi B. Cartwright S. Asherson P. Bolton P. (2015). Brief Report: Adaptive functioning in children with ASD, ADHD and ASD ADHD. *J. Autism Dev. Disord.* 45 2235–2242. 10.1007/s10803-014-2352-y 25614019

[B19] Austerman J. (2015). ADHD and behavioral disorders: Assessment, management, and an update from DSM-5. *Cleve. Clin. J. Med.* 82 S2–S7. 10.3949/ccjm.82.s1.01 26555810

[B20] Avanzino L. Pelosin E. Vicario C. M. Lagravinese G. Abbruzzese G. Martino D. (2016). Time processing and motor control in movement disorders. *Front. Hum. Neurosci.* 10:631. 10.3389/fnhum.2016.00631 28018198 PMC5149591

[B21] Aycicegi-Dinn A. Dervent-Ozbek S. Yazgan Y. Bicer D. Dinn W. M. (2011). Neurocognitive correlates of adult attention-deficit/hyperactivity disorder in a Turkish sample. *Atten. Defic. Hyperact. Disord.* 3 41–52. 10.1007/s12402-010-0050-y 21432617

[B22] Azevedo F. A. C. Carvalho L. R. B. Grinberg L. T. Farfel J. M. Ferretti R. E. L. Leite R. E. P. (2009). Equal numbers of neuronal and nonneuronal cells make the human brain an isometrically scaled-up primate brain. *J. Comp. Neurol.* 513 532–541. 10.1002/cne.21974 19226510

[B23] Baio J. Wiggins L. Christensen D. L. Maenner M. J. Daniels J. Warren Z. (2018). Prevalence of Autism spectrum disorder among children aged 8 Years - Autism and developmental disabilities monitoring network, 11 Sites. United States, 2014. *MMWR Surveill. Summ.* 67 1–23. 10.15585/mmwr.ss6706a1 29701730 PMC5919599

[B24] Barkley R. A. (2010). Differential diagnosis of adults with ADHD: the role of executive function and self-regulation. *J. Clin. Psychiatry* 71:e17. 10.4088/JCP.9066tx1c 20667287

[B25] Barnard L. Muldoon K. Hasan R. O’Brien G. Stewart M. (2008). Profiling executive dysfunction in adults with autism and comorbid learning disability. *Autism* 12 125–141. 10.1177/1362361307088486 18308763

[B26] Barnes J. A. Ebner B. A. Duvick L. A. Gao W. (2011). Abnormalities in the climbing fiber-Purkinje cell circuitry contribute to neuronal dysfunction in ATXN1 [82Q] mice. *J. Neurosci.* 31 12778–12789. 10.1523/JNEUROSCI.2579-11.2011 21900557 PMC3178465

[B27] Bauman M. L. Kemper T. L. (2003). The neuropathology of the autism spectrum disorders: what have we learned? *Novartis Found. Symp.* 251 281–297. 14521190

[B28] Bednarz H. M. Kana R. K. (2019). Patterns of Cerebellar Connectivity with Intrinsic Connectivity Networks in Autism Spectrum Disorders. *J. Autism Dev. Disord.* 49 4498–4514. 10.1007/s10803-019-04168-w 31473949

[B29] Behrmann M. Thomas C. Humphreys K. (2006). Seeing it differently: visual processing in autism. *Trends Cogn. Sci.* 10 258–264. 10.1016/j.tics.2006.05.001 16713326

[B30] Ben-Pazi H. Shalev R. S. Gross-Tsur V. Bergman H. (2006). Age and medication effects on rhythmic responses in ADHD: possible oscillatory mechanisms? *Neuropsychologia* 44 412–416. 10.1016/j.neuropsychologia.2005.05.022 16083921

[B31] Bernard J. A. Mittal V. A. (2014). Cerebellar-motor dysfunction in schizophrenia and psychosis-risk: the importance of regional cerebellar analysis approaches. *Front. Psychiatry* 5:160. 10.3389/fpsyt.2014.00160 25505424 PMC4243486

[B32] Berquin P. C. Giedd J. N. Jacobsen L. K. Hamburger S. D. Krain A. L. Rapoport J. L. (1998). Cerebellum in attention-deficit hyperactivity disorder: a morphometric MRI study. *Neurology* 50 1087–1093. 10.1212/WNL.50.4.1087 9566399

[B33] Bhandari J. Thada P. K. Samanta D. (2022). *“Spinocerebellar Ataxia,” in StatPearls.* Treasure Island, FL: StatPearls Publishing. 32491748

[B34] Biederman J. Lanier J. DiSalvo M. Noyes E. Fried R. Woodworth K. Y. (2019). Clinical correlates of mind wandering in adults with ADHD. *J. Psychiatr. Res.* 117 15–23. 10.1016/j.jpsychires.2019.06.012 31272014

[B35] Bledsoe J. C. Semrud-Clikeman M. Pliszka S. R. (2011). Neuroanatomical and neuropsychological correlates of the cerebellum in children with attention-deficit/hyperactivity disorder–combined type. *J. Am. Acad. Child Adolesc. Psychiatry* 50 593–601. 10.1016/j.jaac.2011.02.014 21621143 PMC3104210

[B36] Boonstra A. M. Oosterlaan J. Sergeant J. A. Buitelaar J. K. (2005). Executive functioning in adult ADHD: a meta-analytic review. *Psychol. Med.* 35 1097–1108. 10.1017/S003329170500499X 16116936

[B37] Bostan A. C. Dum R. P. Strick P. L. (2013). Cerebellar networks with the cerebral cortex and basal ganglia. *Trends Cogn. Sci.* 17 241–254. 10.1016/j.tics.2013.03.003 23579055 PMC3645327

[B38] Bozzi Y. Provenzano G. Casarosa S. (2018). Neurobiological bases of autism-epilepsy comorbidity: a focus on excitation/inhibition imbalance. *Eur. J. Neurosci.* 47 534–548. 10.1111/ejn.13595 28452083

[B39] Bracha V. Zbarska S. Parker K. Carrel A. Zenitsky G. Bloedel J. R. (2009). The cerebellum and eye-blink conditioning: learning versus network performance hypotheses. *Neuroscience* 162 787–796. 10.1016/j.neuroscience.2008.12.042 19162131 PMC2822538

[B40] Braconnier M. L. Siper P. M. (2021). Neuropsychological Assessment in Autism Spectrum Disorder. *Curr. Psychiatry Rep.* 23:63. 10.1007/s11920-021-01277-1 34331144 PMC8324442

[B41] Braga-Neto P. Pedroso J. L. Alessi H. Dutra L. A. Felício A. C. Minett T. (2012). Cerebellar cognitive affective syndrome in Machado Joseph disease: core clinical features. *Cerebellum* 11 549–556. 10.1007/s12311-011-0318-6 21975858

[B42] Brattberg G. (2006). PTSD and ADHD: underlying factors in many cases of burnout. *Stress Health* 22 305–313. 10.1002/smi.1112

[B43] Brodal P. (1978). Principles of organization of the monkey corticopontine projection. *Brain Res.* 148 214–218. 10.1016/0006-8993(78)90392-X 418849

[B44] Bruchhage M. M. K. Bucci M.-P. Becker E. B. E. (2018). Cerebellar involvement in autism and ADHD. *Handb. Clin. Neurol.* 155 61–72. 10.1016/B978-0-444-64189-2.00004-4 29891077

[B45] Buderath P. Gärtner K. Frings M. Christiansen H. Schoch B. Konczak J. (2009). Postural and gait performance in children with attention deficit/hyperactivity disorder. *Gait Posture* 29 249–254. 10.1016/j.gaitpost.2008.08.016 18963991

[B46] Bürk K. Globas C. Bösch S. Klockgether T. Zühlke C. Daum I. (2003). Cognitive deficits in spinocerebellar ataxia type 1, 2, and 3. *J. Neurol.* 250 207–211. 10.1007/s00415-003-0976-5 12574952

[B47] Burright E. N. Clark H. B. Servadio A. Matilla T. Feddersen R. M. Yunis W. S. (1995). SCA1 transgenic mice: a model for neurodegeneration caused by an expanded CAG trinucleotide repeat. *Cell* 82 937–948. 10.1016/0092-8674(95)90273-2 7553854

[B48] Butzbach M. Fuermaier A. B. M. Aschenbrenner S. Weisbrod M. Tucha L. Tucha O. (2019). Basic processes as foundations of cognitive impairment in adult ADHD. *J. Neural Transm.* 126 1347–1362. 10.1007/s00702-019-02049-1 31321549 PMC6764934

[B49] Cai R. Y. Richdale A. L. Uljarević M. Dissanayake C. Samson A. C. (2018). Emotion regulation in autism spectrum disorder: Where we are and where we need to go. *Autism Res.* 11 962–978. 10.1002/aur.1968 29979494

[B50] Callahan B. L. Bierstone D. Stuss D. T. Black S. E. (2017). Adult ADHD: Risk Factor for Dementia or Phenotypic Mimic? *Front. Aging Neurosci.* 9:260. 10.3389/fnagi.2017.00260 28824421 PMC5540971

[B51] Callahan B. L. Ramakrishnan N. Shammi P. Bierstone D. Taylor R. Ozzoude M. (2022). Cognitive and neuroimaging profiles of older adults with attention deficit/hyperactivity disorder presenting to a memory clinic. *J. Atten. Disord.* 26 1118–1129. 10.1177/10870547211060546 34784815 PMC9066671

[B52] Castellanos F. X. Giedd J. N. Marsh W. L. Hamburger S. D. Vaituzis A. C. Dickstein D. P. (1996). Quantitative brain magnetic resonance imaging in attention-deficit hyperactivity disorder. *Arch. Gen. Psychiatry* 53 607–616. 10.1001/archpsyc.1996.01830070053009 8660127

[B53] Castellanos F. X. Lee P. P. Sharp W. Jeffries N. O. Greenstein D. K. Clasen L. S. (2002). Developmental trajectories of brain volume abnormalities in children and adolescents with attention-deficit/hyperactivity disorder. *JAMA* 288 1740–1748. 10.1001/jama.288.14.1740 12365958

[B54] Chen Q. Hartman C. A. Haavik J. Harro J. Klungsøyr K. Hegvik T.-A. (2018). Common psychiatric and metabolic comorbidity of adult attention-deficit/hyperactivity disorder: A population-based cross-sectional study. *PLoS One* 13:e0204516. 10.1371/journal.pone.0204516 30256837 PMC6157884

[B55] Chen X. Tang T.-S. Tu H. Nelson O. Pook M. Hammer R. (2008). Deranged calcium signaling and neurodegeneration in spinocerebellar ataxia type 3. *J. Neurosci.* 28 12713–12724. 10.1523/JNEUROSCI.3909-08.2008 19036964 PMC2663415

[B56] Cheung P. P. P. Lau B. W. M. (2020). Neurobiology of sensory processing in autism spectrum disorder. *Prog. Mol. Biol. Transl. Sci.* 173 161–181. 10.1016/bs.pmbts.2020.04.020 32711809

[B57] Chung S. Son J.-W. (2020). Visual perception in Autism Spectrum Disorder: A review of neuroimaging studies. *Soa Chongs. Chong. Uihak* 31 105–120. 10.5765/jkacap.200018 32665755 PMC7350544

[B58] Cortese S. Castellanos F. X. (2012). Neuroimaging of attention-deficit/hyperactivity disorder: current neuroscience-informed perspectives for clinicians. *Curr. Psychiatry Rep.* 14 568–578. 10.1007/s11920-012-0310-y 22851201 PMC3876939

[B59] Crunelle C. L. Veltman D. J. van Emmerik-van Oortmerssen K. Booij J. van den Brink W. (2013). Impulsivity in adult ADHD patients with and without cocaine dependence. *Drug Alcohol Depend.* 129 18–24. 10.1016/j.drugalcdep.2012.09.006 23026814

[B60] Cumyn L. French L. Hechtman L. (2009). Comorbidity in adults with attention-deficit hyperactivity disorder. *Can. J. Psychiatry* 54 673–683. 10.1177/070674370905401004 19835674

[B61] D’Abreu A. França M. C. Jr. Yasuda C. L. Campos B. A. G. Lopes-Cendes I. Cendes F. (2012). Neocortical atrophy in Machado-Joseph disease: a longitudinal neuroimaging study. *J. Neuroimaging* 22 285–291. 10.1111/j.1552-6569.2011.00614.x 21699609

[B62] Dawson M. Soulières I. Gernsbacher M. A. Mottron L. (2007). The level and nature of autistic intelligence. *Psychol. Sci.* 18 657–662. 10.1111/j.1467-9280.2007.01954.x 17680932 PMC4287210

[B63] De Smet H. J. Paquier P. Verhoeven J. Mariën P. (2013). The cerebellum: its role in language and related cognitive and affective functions. *Brain Lang.* 127 334–342. 10.1016/j.bandl.2012.11.001 23333152

[B64] de Vries M. Geurts H. (2015). Influence of Autism traits and executive functioning on quality of life in children with an Autism spectrum disorder. *J. Autism Dev. Disord.* 45 2734–2743. 10.1007/s10803-015-2438-1 25835211 PMC4553152

[B65] Deberdt W. Thome J. Lebrec J. Kraemer S. Fregenal I. Ramos-Quiroga J. A. (2015). Prevalence of ADHD in nonpsychotic adult psychiatric care (ADPSYC): A multinational cross-sectional study in Europe. *BMC Psychiatry* 15:242. 10.1186/s12888-015-0624-5 26462666 PMC4604704

[B66] Demetriou E. A. Lampit A. Quintana D. S. Naismith S. L. Song Y. J. C. Pye J. E. (2018). Autism spectrum disorders: a meta-analysis of executive function. *Mol. Psychiatry* 23 1198–1204. 10.1038/mp.2017.75 28439105 PMC5984099

[B67] Depue B. E. Burgess G. C. Willcutt E. G. Bidwell L. C. Ruzic L. Banich M. T. (2010). Symptom-correlated brain regions in young adults with combined-type ADHD: their organization, variability, and relation to behavioral performance. *Psychiatry Res.* 182 96–102. 10.1016/j.pscychresns.2009.11.011 20399622 PMC2878757

[B68] Dickson P. E. Rogers T. D. Del Mar N. Martin L. A. Heck D. Blaha C. D. (2010). Behavioral flexibility in a mouse model of developmental cerebellar Purkinje cell loss. *Neurobiol. Learn. Mem.* 94 220–228. 10.1016/j.nlm.2010.05.010 20566377 PMC2922405

[B69] Diedrichsen J. King M. Hernandez-Castillo C. Sereno M. Ivry R. B. (2019). Universal transform or multiple functionality? Understanding the contribution of the human cerebellum across task domains. *Neuron* 102 918–928. 10.1016/j.neuron.2019.04.021 31170400 PMC6686189

[B70] Dirks H. Scherbaum N. Kis B. Mette C. (2017). ADHD in Adults and Comorbid Substance Use Disorder: Prevalence, Clinical Diagnostics and Integrated Therapy. *Fortschr. Neurol. Psychiatr.* 85 336–344. 10.1055/s-0043-100763 28645126

[B71] D’Mello A. M. Crocetti D. Mostofsky S. H. Stoodley C. J. (2015). Cerebellar gray matter and lobular volumes correlate with core autism symptoms. *NeuroImage* 7 631–639. 10.1016/j.nicl.2015.02.007 25844317 PMC4375648

[B72] Doernberg E. Hollander E. (2016). Neurodevelopmental Disorders (ASD and ADHD): DSM-5, ICD-10, and ICD-11. *CNS Spectr.* 21 295–299. 10.1017/S1092852916000262 27364515

[B73] Doi H. Kanai C. Ohta H. (2022). Transdiagnostic and sex differences in cognitive profiles of autism spectrum disorder and attention-deficit/hyperactivity disorder. *Autism Res.* 15 1130–1141. 10.1002/aur.2712 35347878

[B74] Downs A. Downs R. C. Rau K. (2008). Effects of training and feedback on Discrete Trial Teaching skills and student performance. *Res. Dev. Disabil.* 29 235–246. 10.1016/j.ridd.2007.05.001 17582740

[B75] Duan K. Jiang W. Rootes-Murdy K. Schoenmacker G. H. Arias-Vasquez A. Buitelaar J. K. (2021). Gray matter networks associated with attention and working memory deficit in ADHD across adolescence and adulthood. *Transl. Psychiatry* 11:184. 10.1038/s41398-021-01301-1 33767139 PMC7994833

[B76] Durr A. (2010). Autosomal dominant cerebellar ataxias: polyglutamine expansions and beyond. *Lancet Neurol.* 9 885–894. 10.1016/S1474-4422(10)70183-6 20723845

[B77] E K.-H. Chen S.-H. A. Ho M.-H. R. Desmond J. E. (2014). A meta-analysis of cerebellar contributions to higher cognition from PET and fMRI studies. *Hum. Brain Mapp.* 35 593–615. 10.1002/hbm.22194 23125108 PMC3866223

[B78] El Malhany N. Gulisano M. Rizzo R. Curatolo P. (2015). Tourette syndrome and comorbid ADHD: causes and consequences. *Eur. J. Pediatr.* 174 279–288. 10.1007/s00431-014-2417-0 25224657

[B79] Eliassen J. C. Souza T. Sanes J. N. (2001). Human brain activation accompanying explicitly directed movement sequence learning. *Exp. Brain Res.* 141 269–280. 10.1007/s002210100822 11715072

[B80] Elyoseph Z. Mintz M. Vakil E. Zaltzman R. Gordon C. R. (2020). Selective Procedural Memory Impairment but Preserved Declarative Memory in Spinocerebellar Ataxia Type 3. *Cerebellum* 19 226–234. 10.1007/s12311-019-01101-w 31912433

[B81] Enticott P. G. Kennedy H. A. Johnston P. J. Rinehart N. J. Tonge B. J. Taffe J. R. (2014). Emotion recognition of static and dynamic faces in autism spectrum disorder. *Cogn. Emot.* 28 1110–1118. 10.1080/02699931.2013.867832 24341852

[B82] Esbensen A. J. Seltzer M. M. Lam K. S. L. Bodfish J. W. (2009). Age-related differences in restricted repetitive behaviors in autism spectrum disorders. *J. Autism Dev. Disord.* 39 57–66. 10.1007/s10803-008-0599-x 18566881 PMC2605515

[B83] Fabio R. A. Caprì T. (2017). The executive functions in a sample of Italian adults with ADHD: attention, response inhibition and planning/organization. *Mediter. J. Clin. Psychology* 5 1–17.

[B84] Fadeuilhe C. Daigre C. Richarte V. Grau-López L. Palma-Álvarez R. F. Corrales M. (2021). Insomnia Disorder in Adult Attention-Deficit/Hyperactivity Disorder Patients: Clinical. Comorbidity, and Treatment Correlates. *Front. Psychiatry* 12:663889. 10.3389/fpsyt.2021.663889 34122179 PMC8187558

[B85] Fastenrath M. Spalek K. Coynel D. Loos E. Milnik A. Egli T. (2022). Human cerebellum and corticocerebellar connections involved in emotional memory enhancement. *Proc. Natl. Acad. Sci. U. S. A.* 119:e2204900119. 10.1073/pnas.2204900119 36191198 PMC9564100

[B86] Feng L. Chen D. B. Hou L. Huang L. H. Lu S. Y. Liang X. L. (2014). Cognitive impairment in native Chinese with spinocerebellar ataxia type 3. *Eur. Neurol.* 71 262–270. 10.1159/000357404 24525517

[B87] Fernandez L. Burmester A. Duque J. D. Silk T. J. Hyde C. E. Kirkovski M. (2022). Examination of Cerebellar Grey-Matter Volume in Children with Neurodevelopmental Disorders: a Coordinated Analysis Using the ACAPULCO Algorithm. *Cerebellum* [Epub ahead of print]. 10.1007/s12311-022-01503-3 36482028

[B88] Fine E. J. Ionita C. C. Lohr L. (2002). The history of the development of the cerebellar examination. *Semin. Neurol.* 22 375–384. 10.1055/s-2002-36759 12539058

[B89] Fisher J. D. T. (1839). ART. VII. Contributions Illustrative of the Functions of the Cerebellum. *Am. J. Med. Sci.* 23:1827.

[B90] Fletcher F. E. Knowland V. Walker S. Gaskell M. G. Norbury C. Henderson L. M. (2020). Atypicalities in sleep and semantic consolidation in autism. *Dev. Sci.* 23:e12906. 10.1111/desc.12906 31569286 PMC7187235

[B91] Flourens P. (1824). *Experimental research on the properties and functions of the nervous system in vertebrate animals.* Paris: Crevot. PMC10463104 38080577

[B92] Freedman E. G. Foxe J. J. (2018). Eye movements, sensorimotor adaptation and cerebellar-dependent learning in autism: toward potential biomarkers and subphenotypes. *Eur. J. Neurosci.* 47 549–555. 10.1111/ejn.13625 28612953 PMC11800192

[B93] Freeman L. M. Locke J. Rotheram-Fuller E. Mandell D. (2017). Brief Report: Examining Executive and Social Functioning in Elementary-Aged Children with Autism. *J. Autism Dev. Disord.* 47 1890–1895. 10.1007/s10803-017-3079-3 28260182 PMC5536167

[B94] Frings M. Gaertner K. Buderath P. Gerwig M. Christiansen H. Schoch B. (2010). Timing of conditioned eyeblink responses is impaired in children with attention-deficit/hyperactivity disorder. *Exp. Brain Res.* 201 167–176. 10.1007/s00221-009-2020-1 19777220

[B95] Fusar-Poli P. Placentino A. Carletti F. Landi P. Allen P. Surguladze S. (2009). Functional atlas of emotional faces processing: a voxel-based meta-analysis of 105 functional magnetic resonance imaging studies. *J. Psychiatry Neurosci.* 34 418–432. 19949718 PMC2783433

[B96] Garrard P. Martin N. H. Giunti P. Cipolotti L. (2008). Cognitive and social cognitive functioning in spinocerebellar ataxia: a preliminary characterization. *J. Neurol.* 255 398–405. 10.1007/s00415-008-0680-6 18350360

[B97] Gatti D. Vecchi T. Mazzoni G. (2021). Cerebellum and semantic memory: A TMS study using the DRM paradigm. *Cortex* 135 78–91. 10.1016/j.cortex.2020.11.017 33360762

[B98] Gellersen H. M. Guo C. C. O’Callaghan C. Tan R. H. Sami S. Hornberger M. (2017). Cerebellar atrophy in neurodegeneration-a meta-analysis. *J. Neurol. Neurosurg. Psychiatry* 88 780–788. 10.1136/jnnp-2017-315607 28501823

[B99] Gerwig M. Kolb F. P. Timmann D. (2007). The involvement of the human cerebellum in eyeblink conditioning. *Cerebellum* 6 38–57. 10.1080/14734220701225904 17366265

[B100] Geurts H. M. Verté S. Oosterlaan J. Roeyers H. Sergeant J. A. (2004). How specific are executive functioning deficits in attention deficit hyperactivity disorder and autism? *J. Child Psychol. Psychiatry* 45 836–854. 10.1111/j.1469-7610.2004.00276.x 15056314

[B101] Giedd J. N. (2019). The Enigma of Neuroimaging in ADHD. *Am. J. Psychiatry* 176 503–504. 10.1176/appi.ajp.2019.19050540 31256625

[B102] Gilbert S. J. Bird G. Brindley R. Frith C. D. Burgess P. W. (2008). Atypical recruitment of medial prefrontal cortex in autism spectrum disorders: an fMRI study of two executive function tasks. *Neuropsychologia* 46 2281–2291. 10.1016/j.neuropsychologia.2008.03.025 18485420 PMC2648877

[B103] Gilchrist A. Green J. Cox A. Burton D. Rutter M. Le Couteur A. (2001). Development and current functioning in adolescents with Asperger syndrome: a comparative study. *J. Child Psychol. Psychiatry* 42 227–240. 10.1111/1469-7610.00714 11280419

[B104] Gillberg C. Rasmussen P. (2011). “Neuropsykiatriska funktionsnedsättningar med debut i tidig barndom,” in *Kognitiv Medicin*, ed. Lars-Olof Wahlund C. N. A. A. W. (Swedish: Norstedts), 160–182.

[B105] Gold J. M. Carpenter C. Randolph C. Goldberg T. E. Weinberger D. R. (1997). Auditory working memory and Wisconsin Card Sorting Test performance in schizophrenia. *Arch. Gen. Psychiatry* 54 159–165. 10.1001/archpsyc.1997.01830140071013 9040284

[B106] Goldberg M. C. Mostofsky S. H. Cutting L. E. Mahone E. M. Astor B. C. Denckla M. B. (2005). Subtle executive impairment in children with autism and children with ADHD. *J. Autism Dev. Disord.* 35 279–293. 10.1007/s10803-005-3291-4 16119469

[B107] Griffin J. W. Bauer R. Gavett B. E. (2022). The Episodic Memory Profile in Autism Spectrum Disorder: A Bayesian Meta-Analysis. *Neuropsychol. Rev.* 32 316–351. 10.1007/s11065-021-09493-5 33954915

[B108] Griffiths S. Jarrold C. Penton-Voak I. S. Woods A. T. Skinner A. L. Munafò M. R. (2019). Impaired Recognition of Basic Emotions from Facial Expressions in Young People with Autism Spectrum Disorder: Assessing the Importance of Expression Intensity. *J. Autism Dev. Disord.* 49 2768–2778. 10.1007/s10803-017-3091-7 28361375 PMC6606653

[B109] Grill J. Viguier D. Kieffer V. Bulteau C. Sainte-Rose C. Hartmann O. (2004). Critical risk factors for intellectual impairment in children with posterior fossa tumors: the role of cerebellar damage. *J. Neurosurg.* 101 152–158. 10.3171/ped.2004.101.2.0152 15835102

[B110] Guo N. Fuermaier A. B. M. Koerts J. Mueller B. W. Diers K. Mroß A. (2021). Neuropsychological functioning of individuals at clinical evaluation of adult ADHD. *J. Neural Transm.* 128 877–891. 10.1007/s00702-020-02281-0 33355692 PMC8295106

[B111] Guy J. Mottron L. Berthiaume C. Bertone A. (2019). A Developmental Perspective of Global and Local Visual Perception in Autism Spectrum Disorder. *J. Autism Dev. Disord.* 49 2706–2720. 10.1007/s10803-016-2834-1 27371139

[B112] Hansen S. T. Meera P. Otis T. S. Pulst S. M. (2013). Changes in Purkinje cell firing and gene expression precede behavioral pathology in a mouse model of SCA2. *Hum. Mol. Genet.* 22 271–283. 10.1093/hmg/dds427 23087021 PMC3526159

[B113] Happé F. Ronald A. Plomin R. (2006). Time to give up on a single explanation for autism. *Nat. Neurosci.* 9 1218–1220. 10.1038/nn1770 17001340

[B114] Hartmann-von Monakow K. Akert K. Künzle H. (1981). Projection of precentral, premotor and prefrontal cortex to the basilar pontine grey and to nucleus reticularis tegmenti pontis in the monkey (Macaca fascicularis). *Schweiz. Arch. Neurol. Neurochir. Psychiatr.* 129 189–208. 7323772

[B115] Hegerl U. Himmerich H. Engmann B. Hensch T. (2010). Mania and attention-deficit/hyperactivity disorder: common symptomatology, common pathophysiology and common treatment? *Curr. Opin. Psychiatry* 23 1–7. 10.1097/YCO.0b013e328331f694 19770771

[B116] Hervey A. S. Epstein J. N. Curry J. F. (2004). Neuropsychology of adults with attention-deficit/hyperactivity disorder: a meta-analytic review. *Neuropsychology* 18 485–503. 10.1037/0894-4105.18.3.485 15291727

[B117] Hesslow G. Yeo C. H. (2002). “The Functional Anatomy of Skeletal Conditioning,” in *A Neuroscientist’s Guide to Classical Conditioning*, ed. Moore J. W. (New York, NY: Springer New York), 86–146. 10.1007/978-1-4419-8558-3_4

[B118] Hill E. L. (2004). Executive dysfunction in autism. *Trends Cogn. Sci.* 8 26–32. 10.1016/j.tics.2003.11.003 14697400

[B119] Hintzen A. Pelzer E. A. Tittgemeyer M. (2018). Thalamic interactions of cerebellum and basal ganglia. *Brain Struct. Funct.* 223 569–587. 10.1007/s00429-017-1584-y 29224175

[B120] Hirose S. Jimura K. Kunimatsu A. Abe O. Ohtomo K. Miyashita Y. (2014). Changes in cerebro-cerebellar interaction during response inhibition after performance improvement. *Neuroimage* 99 142–148. 10.1016/j.neuroimage.2014.05.007 24830836

[B121] Hlavatá P. Kašpárek T. Linhartová P. Ošlejšková H. Bareš M. (2018). Autism, impulsivity and inhibition a review of the literature. *Basal Ganglia* 14 44–53. 10.1016/j.baga.2018.10.002 11280926

[B122] Hoche F. Guell X. Sherman J. C. Vangel M. G. Schmahmann J. D. (2016). Cerebellar Contribution to Social Cognition. *Cerebellum* 15 732–743. 10.1007/s12311-015-0746-9 26585120 PMC5157127

[B123] Hodges H. Fealko C. Soares N. (2020). Autism spectrum disorder: definition, epidemiology, causes, and clinical evaluation. *Transl. Pediatr.* 9 S55–S65. 10.21037/tp.2019.09.09 32206584 PMC7082249

[B124] Holdefer R. N. Miller L. E. Chen L. L. Houk J. C. (2000). Functional connectivity between cerebellum and primary motor cortex in the awake monkey. *J. Neurophysiol.* 84 585–590. 10.1152/jn.2000.84.1.585 10899231

[B125] Homack S. Lee D. Riccio C. A. (2005). Test review: Delis-Kaplan executive function system. *J. Clin. Exp. Neuropsychol.* 27 599–609. 10.1080/13803390490918444 16019636

[B126] Hourez R. Servais L. Orduz D. Gall D. Millard I. de Kerchove (2011). Aminopyridines correct early dysfunction and delay neurodegeneration in a mouse model of spinocerebellar ataxia type 1. *J. Neurosci.* 31 11795–11807. 10.1523/JNEUROSCI.0905-11.2011 21849540 PMC6623197

[B127] Hoxha E. Balbo I. Miniaci M. C. Tempia F. (2018). Purkinje Cell Signaling Deficits in Animal Models of Ataxia. *Front. Synaptic Neurosci.* 10:6. 10.3389/fnsyn.2018.00006 29760657 PMC5937225

[B128] Hughes C. Russell J. Robbins T. W. (1994). Evidence for executive dysfunction in autism. *Neuropsychologia* 32 477–492. 10.1016/0028-3932(94)90092-2 8047253

[B129] Hyde K. L. Samson F. Evans A. C. Mottron L. (2010). Neuroanatomical differences in brain areas implicated in perceptual and other core features of autism revealed by cortical thickness analysis and voxel-based morphometry. *Hum. Brain Mapp.* 31 556–566. 10.1002/hbm.20887 19790171 PMC6870833

[B130] Ivry R. B. Keele S. W. (1989). Timing functions of the cerebellum. *J. Cogn. Neurosci.* 1 136–152. 10.1162/jocn.1989.1.2.136 23968462

[B131] Johnson B. P. Rinehart N. J. White O. Millist L. Fielding J. (2013). Saccade adaptation in autism and Asperger’s disorder. *Neuroscience* 243 76–87. 10.1016/j.neuroscience.2013.03.051 23562581

[B132] Johnson C. N. Ramphal B. Koe E. Raudales A. Goldsmith J. Margolis A. E. (2021). Cognitive correlates of autism spectrum disorder symptoms. *Autism Res.* 14 2405–2411. 10.1002/aur.2577 34269525 PMC8578389

[B133] Jones C. R. G. Simonoff E. Baird G. Pickles A. Marsden A. J. S. Tregay J. (2018). The association between theory of mind, executive function, and the symptoms of autism spectrum disorder. *Autism Res.* 11 95–109. 10.1002/aur.1873 28945319

[B134] K B R. Krzyspiak J. Khodakhah K. (2022). Subthalamic nucleus modulation of the pontine nuclei and its targeting of the cerebellar cortex. *J. Neurosci.* [Epub ahead of print]. 10.1523/JNEUROSCI.2388-19.2022 35641185 PMC9295842

[B135] Kamphaus R. W. (2005). *Clinical Assessment of Child and Adolescent Intelligence.* New York, NY: Springer. 10.1007/978-0-387-29149-9_11

[B136] Karatekin C. Lazareff J. A. Asarnow R. F. (2000). Relevance of the cerebellar hemispheres for executive functions. *Pediatr. Neurol.* 22 106–112. 10.1016/S0887-8994(99)00128-9 10738915

[B137] Kasumu A. W. Liang X. Egorova P. Vorontsova D. Bezprozvanny I. (2012). Chronic suppression of inositol 1,4,5-triphosphate receptor-mediated calcium signaling in cerebellar purkinje cells alleviates pathological phenotype in spinocerebellar ataxia 2 mice. *J. Neurosci.* 32 12786–12796. 10.1523/JNEUROSCI.1643-12.2012 22973002 PMC3470884

[B138] Kawai Y. Takeda A. Abe Y. Washimi Y. Tanaka F. Sobue G. (2004). Cognitive impairments in Machado-Joseph disease. *Arch. Neurol.* 61 1757–1760. 10.1001/archneur.61.11.1757 15534186

[B139] Kelly R. M. Strick P. L. (2003). Cerebellar loops with motor cortex and prefrontal cortex of a nonhuman primate. *J. Neurosci.* 23 8432–8444. 10.1523/JNEUROSCI.23-23-08432.2003 12968006 PMC6740694

[B140] Kercood S. Grskovic J. A. Banda D. Begeske J. (2014). Working memory and autism: A review of literature. *Res. Autism Spectr. Disord.* 8 1316–1332. 10.1016/j.rasd.2014.06.011

[B141] Kessler R. C. Green J. G. Adler L. A. Barkley R. A. Chatterji S. Faraone S. V. (2010). Structure and diagnosis of adult attention-deficit/hyperactivity disorder: analysis of expanded symptom criteria from the Adult ADHD Clinical Diagnostic Scale. *Arch. Gen. Psychiatry* 67 1168–1178. 10.1001/archgenpsychiatry.2010.146 21041618 PMC3131739

[B142] Kim S. E. Jung S. Sung G. Bang M. Lee S.-H. (2021). Impaired cerebro-cerebellar white matter connectivity and its associations with cognitive function in patients with schizophrenia. *npj Schizophrenia* 7 1–7. 10.1038/s41537-021-00169-w 34385473 PMC8360938

[B143] Kim S. G. Uğurbil K. Strick P. L. (1994). Activation of a cerebellar output nucleus during cognitive processing. *Science* 265 949–951. 10.1126/science.8052851 8052851

[B144] King M. Hernandez-Castillo C. R. Poldrack R. A. Ivry R. B. Diedrichsen J. (2019). Functional boundaries in the human cerebellum revealed by a multi-domain task battery. *Nat. Neurosci.* 22 1371–1378. 10.1038/s41593-019-0436-x 31285616 PMC8312478

[B145] Kis B. Guberina N. Kraemer M. Niklewski F. Dziobek I. Wiltfang J. (2017). Perception of emotional prosody in adults with attention deficit hyperactivity disorder. *Acta Psychiatr. Scand.* 135 506–514. 10.1111/acps.12719 28276052

[B146] Klinke I. Minnerop M. Schmitz-Hübsch T. Hendriks M. Klockgether T. Wüllner U. (2010). Neuropsychological features of patients with spinocerebellar ataxia (SCA) types 1, 2, 3, and 6. *Cerebellum* 9 433–442. 10.1007/s12311-010-0183-8 20502998 PMC2949561

[B147] Klockgether T. (2010). Sporadic ataxia with adult onset: classification and diagnostic criteria. *Lancet Neurol.* 9 94–104. 10.1016/S1474-4422(09)70305-9 20083040

[B148] Koltermann G. Becker N. Wauke A. P. T. de Oliveira C. P. Gomides M. R. de A. (2020). Intragroup differences and similarities in performance on rapid automatized naming tasks in children with ADHD symptoms, children with reading disabilities, and controls. *Trends Psychiatry Psychother* 42 190–194. 10.1590/2237-6089-2019-0014 32520167

[B149] Kucyi A. Hove M. J. Biederman J. Van Dijk K. R. A. Valera E. M. (2015). Disrupted functional connectivity of cerebellar default network areas in attention-deficit/hyperactivity disorder. *Hum. Brain Mapp.* 36 3373–3386. 10.1002/hbm.22850 26109476 PMC4562390

[B150] Kuo S.-H. (2019). Ataxia. *Continuum* 25 1036–1054. 10.1212/CON.0000000000000753 31356292 PMC7339377

[B151] La Malfa G. Lassi S. Bertelli M. Salvini R. Placidi G. F. (2004). Autism and intellectual disability: a study of prevalence on a sample of the Italian population. *J. Intellect. Disabil. Res.* 48 262–267. 10.1111/j.1365-2788.2003.00567.x 15025669

[B152] Lai M.-C. Lombardo M. V. Baron-Cohen S. (2014). Autism. *The Lancet* 383 896–910. 10.1016/S0140-6736(13)61539-1 24074734

[B153] Lalonde R. Botez-Marquard T. (2000). Neuropsychological deficits of patients with chronic or acute cerebellar lesions. *J. Neurolinguistics* 13 117–128. 10.1016/S0911-6044(00)00007-5

[B154] Landa R. J. Goldberg M. C. (2005). Language, social, and executive functions in high functioning autism: a continuum of performance. *J. Autism Dev. Disord.* 35 557–573. 10.1007/s10803-005-0001-1 16211332

[B155] Leggio M. G. Silveri M. C. Petrosini L. Molinari M. (2000). Phonological grouping is specifically affected in cerebellar patients: a verbal fluency study. *J. Neurol. Neurosurg. Psychiatry* 69 102–106. 10.1136/jnnp.69.1.102 10864613 PMC1736989

[B156] Leitner Y. (2014). The co-occurrence of autism and attention deficit hyperactivity disorder in children - What Do We Know? *Front. Hum. Neurosci.* 8:268. 10.3389/fnhum.2014.00268 24808851 PMC4010758

[B157] Lindsay E. Storey E. (2017). Cognitive Changes in the Spinocerebellar Ataxias Due to Expanded Polyglutamine Tracts: A Survey of the Literature. *Brain Sci.* 7:83. 10.3390/brainsci7070083 28708110 PMC5532596

[B158] Liu J. Tang T.-S. Tu H. Nelson O. Herndon E. Huynh D. P. (2009). Deranged calcium signaling and neurodegeneration in spinocerebellar ataxia type 2. *J. Neurosci.* 29 9148–9162. 10.1523/JNEUROSCI.0660-09.2009 19625506 PMC2749883

[B159] Lopes T. M. D’Abreu A. França M. C. Jr. Yasuda C. L. Betting L. E. Samara A. B. (2013). Widespread neuronal damage and cognitive dysfunction in spinocerebellar ataxia type 3. *J. Neurol.* 260 2370–2379. 10.1007/s00415-013-6998-8 23775343

[B160] Luders E. Narr K. L. Hamilton L. S. Phillips O. R. Thompson P. M. Valle J. S. (2009). Decreased callosal thickness in attention-deficit/hyperactivity disorder. *Biol. Psychiatry* 65 84–88. 10.1016/j.biopsych.2008.08.027 18842255 PMC2773144

[B161] Ma J. Wu C. Lei J. Zhang X. (2014). Cognitive impairments in patients with spinocerebellar ataxia types 1, 2 and 3 are positively correlated to the clinical severity of ataxia symptoms. *Int. J. Clin. Exp. Med.* 7 5765–5771. 25664104 PMC4307551

[B162] Maas R. P. P. W. M. Killaars S. van de Warrenburg B. P. C. Schutter D. J. L. G. (2021). The cerebellar cognitive affective syndrome scale reveals early neuropsychological deficits in SCA3 patients. *J. Neurol.* 268 3456–3466. 10.1007/s00415-021-10516-7 33743045 PMC8357713

[B163] Mackie S. Shaw P. Lenroot R. Pierson R. Greenstein D. K. Nugent T. F. (2007). Cerebellar development and clinical outcome in attention deficit hyperactivity disorder. *AJP* 164 647–655. 10.1176/ajp.2007.164.4.647 17403979

[B164] MacLeod C. M. (1991). Half a century of research on the Stroop effect: an integrative review. *Psychol. Bull.* 109 163–203. 10.1037/0033-2909.109.2.163 2034749

[B165] Manes F. Villamil A. R. Ameriso S. Roca M. Torralva T. (2009). “Real life” executive deficits in patients with focal vascular lesions affecting the cerebellum. *J. Neurol. Sci.* 283 95–98. 10.1016/j.jns.2009.02.316 19268308

[B166] Manto M. Bower J. M. Conforto A. B. Delgado-García J. M. da Guarda S. N. F. Gerwig M. (2012). Consensus Paper: Roles of the Cerebellum in Motor Control—The Diversity of Ideas on Cerebellar Involvement in Movement. *Cerebellum* 11 457–487. 10.1007/s12311-011-0331-9 22161499 PMC4347949

[B167] Mariën P. Ackermann H. Adamaszek M. Barwood C. H. S. Beaton A. Desmond J. (2014). Consensus paper: Language and the cerebellum: an ongoing enigma. *Cerebellum* 13 386–410. 10.1007/s12311-013-0540-5 24318484 PMC4090012

[B168] Mariën P. Borgatti R. (2018). Language and the cerebellum. *Handb. Clin. Neurol.* 154 181–202. 10.1016/B978-0-444-63956-1.00011-4 29903439

[B169] Marinopoulou M. Lugnegård T. Hallerbäck M. U. Gillberg C. Billstedt E. (2016). Asperger Syndrome and Schizophrenia: A Comparative Neuropsychological Study. *J. Autism Dev. Disord.* 46 2292–2304. 10.1007/s10803-016-2758-9 26936160

[B170] Martin L. A. Goldowitz D. Mittleman G. (2010). Repetitive behavior and increased activity in mice with Purkinje cell loss: a model for understanding the role of cerebellar pathology in autism. *Eur. J. Neurosci.* 31 544–555. 10.1111/j.1460-9568.2009.07073.x 20105240 PMC2846709

[B171] Matson J. L. Shoemaker M. (2009). Intellectual disability and its relationship to autism spectrum disorders. *Res. Dev. Disabil.* 30 1107–1114. 10.1016/j.ridd.2009.06.003 19604668

[B172] McDougle S. D. Tsay J. S. Pitt B. King M. Saban W. Taylor J. A. (2022). Continuous manipulation of mental representations is compromised in cerebellar degeneration. *Brain* [Epub ahead of print]. 10.1093/brain/awac072 35202465 PMC10200308

[B173] Meyers J. E. Kr M. (1995). *Rey Complex Figure Test and Recognition Trial.* Odessa, TX: Psychological Assessment Resources.

[B174] Middleton F. A. Strick P. L. (1994). Anatomical evidence for cerebellar and basal ganglia involvement in higher cognitive function. *Science* 266 458–461. 10.1126/science.7939688 7939688

[B175] Miniscalco C. Nygren G. Hagberg B. Kadesjö B. Gillberg C. (2006). Neuropsychiatric and neurodevelopmental outcome of children at age 6 and 7 years who screened positive for language problems at 30 months. *Dev. Med. Child Neurol.* 48 361–366. 10.1017/S0012162206000788 16608544

[B176] Minshew N. J. Goldstein G. Siegel D. J. (1997). Neuropsychologic functioning in autism: profile of a complex information processing disorder. *J. Int. Neuropsychol. Soc.* 3 303–316. 10.1017/S1355617797003032 9260440

[B177] Mittleman G. Goldowitz D. Heck D. H. Blaha C. D. (2008). Cerebellar modulation of frontal cortex dopamine efflux in mice: relevance to autism and schizophrenia. *Synapse* 62 544–550. 10.1002/syn.20525 18435424 PMC3854870

[B178] Mohamed S. M. H. Butzbach M. Fuermaier A. B. M. Weisbrod M. Aschenbrenner S. Tucha L. (2021). Basic and complex cognitive functions in Adult ADHD. *PLoS One* 16:e0256228. 10.1371/journal.pone.0256228 34473722 PMC8412315

[B179] Morellini L. Ceroni M. Rossi S. Zerboni G. Rege-Colet L. Biglia E. (2022). Social Cognition in Adult ADHD: A Systematic Review. *Front. Psychol.* 13:940445. 10.3389/fpsyg.2022.940445 35898990 PMC9311421

[B180] Moriarty A. Cook A. Hunt H. Adams M. E. Cipolotti L. Giunti P. (2016). A longitudinal investigation into cognition and disease progression in spinocerebellar ataxia types 1, 2, 3, 6, and 7. *Orphanet J. Rare Dis.* 11:82. 10.1186/s13023-016-0447-6 27333979 PMC4917932

[B181] Morimoto C. Hida E. Shima K. Okamura H. (2018). Temporal processing instability with millisecond accuracy is a cardinal feature of sensorimotor impairments in Autism spectrum disorder: analysis using the synchronized finger-tapping task. *J. Autism Dev. Disord.* 48 351–360. 10.1007/s10803-017-3334-7 28988374

[B182] Moro A. Moscovich M. Farah M. Camargo C. H. F. Teive H. A. G. Munhoz R. P. (2019). Nonmotor symptoms in spinocerebellar ataxias (SCAs). *Cerebel. Atax.* 6:12. 10.1186/s40673-019-0106-5 31485334 PMC6712685

[B183] Morrison K. E. DeBrabander K. M. Jones D. R. Ackerman R. A. Sasson N. J. (2020). Social Cognition, Social Skill, and Social Motivation Minimally Predict Social Interaction Outcomes for Autistic and Non-Autistic Adults. *Front. Psychol.* 11:591100. 10.3389/fpsyg.2020.591100 33324295 PMC7723837

[B184] Mostofsky S. H. Powell S. K. Simmonds D. J. Goldberg M. C. Caffo B. Pekar J. J. (2009). Decreased connectivity and cerebellar activity in autism during motor task performance. *Brain* 132 2413–2425. 10.1093/brain/awp088 19389870 PMC2732264

[B185] Mottron L. Burack J. A. (2001). “Enhanced perceptual functioning in the development of autism,” in *The development of autism: Perspectives from theory and research*, ed. Burack J. A. (Mahwah, NJ: Lawrence Erlbaum Associates Publishers), 131–148.

[B186] Mottron L. Dawson M. Soulières I. Hubert B. Burack J. (2006). Enhanced perceptual functioning in autism: an update, and eight principles of autistic perception. *J. Autism Dev. Disord.* 36 27–43. 10.1007/s10803-005-0040-7 16453071

[B187] Nardone S. Elliott E. (2016). The Interaction between the Immune System and Epigenetics in the Etiology of Autism Spectrum Disorders. *Front. Neurosci.* 10:329. 10.3389/fnins.2016.00329 27462204 PMC4940387

[B188] Newschaffer C. J. Fallin D. Lee N. L. (2002). Heritable and nonheritable risk factors for autism spectrum disorders. *Epidemiol. Rev.* 24 137–153. 10.1093/epirev/mxf010 12762089

[B189] Nikolas M. A. Marshall P. Hoelzle J. B. (2019). The role of neurocognitive tests in the assessment of adult attention-deficit/hyperactivity disorder. *Psychol. Assess.* 31 685–698. 10.1037/pas0000688 30730189

[B190] Noens I. L. J. van Berckelaer-Onnes I. A. (2005). Captured by details: sense-making, language and communication in autism. *J. Commun. Disord.* 38 123–141. 10.1016/j.jcomdis.2004.06.002 15571713

[B191] Oristaglio J. Hyman West S. Ghaffari M. Lech M. S. Verma B. R. Harvey J. A. (2013). Children with autism spectrum disorders show abnormal conditioned response timing on delay, but not trace, eyeblink conditioning. *Neuroscience* 248 708–718. 10.1016/j.neuroscience.2013.06.007 23769889 PMC3791861

[B192] Paulson H. (2012). “Machado–Joseph disease/spinocerebellar ataxia type 3,” in *Handbook of Clinical Neurology*, eds Subramony S. H. Dürr A. (Berlin: Elsevier), 437–449. 10.1016/B978-0-444-51892-7.00027-9 PMC3568768 21827905

[B193] Pellicano E. (2007). Links between theory of mind and executive function in young children with autism: clues to developmental primacy. *Dev. Psychol.* 43 974–990. 10.1037/0012-1649.43.4.974 17605529

[B194] Pereira-Sanchez V. Castellanos F. X. (2021). Neuroimaging in attention-deficit/hyperactivity disorder. *Curr. Opin. Psychiatry* 34 105–111. 10.1097/YCO.0000000000000669 33278156 PMC7879851

[B195] Petacchi A. Laird A. R. Fox P. T. Bower J. M. (2005). Cerebellum and auditory function: an ALE meta-analysis of functional neuroimaging studies. *Hum. Brain Mapp.* 25 118–128. 10.1002/hbm.20137 15846816 PMC6871682

[B196] Petersen S. E. Fox P. T. Posner M. I. Mintun M. Raichle M. E. (1988). Positron emission tomographic studies of the cortical anatomy of single-word processing. *Nature* 331 585–589. 10.1038/331585a0 3277066

[B197] Petersen S. E. Fox P. T. Posner M. I. Mintun M. Raichle M. E. (1989). Positron emission tomographic studies of the processing of singe words. *J. Cogn. Neurosci.* 1 153–170. 10.1162/jocn.1989.1.2.153 23968463

[B198] Poroca D. R. Pelis R. M. Chappe V. M. (2017). ClC Channels and Transporters: Structure, Physiological Functions, and Implications in Human Chloride Channelopathies. *Front. Pharmacol.* 8:151. 10.3389/fphar.2017.00151 28386229 PMC5362633

[B199] Qiu M.-G. Ye Z. Li Q.-Y. Liu G.-J. Xie B. Wang J. (2011). Changes of brain structure and function in ADHD children. *Brain Topogr.* 24 243–252. 10.1007/s10548-010-0168-4 21191807

[B200] Ray K. L. Ragland J. D. MacDonald A. W. Gold J. M. Silverstein S. M. Barch D. M. (2020). Dynamic reorganization of the frontal parietal network during cognitive control and episodic memory. *Cogn. Affect. Behav. Neurosci.* 20 76–90. 10.3758/s13415-019-00753-9 31811557 PMC7018593

[B201] Reeber S. L. Otis T. S. Sillitoe R. V. (2013). New roles for the cerebellum in health and disease. *Front. Syst. Neurosci.* 7:83. 10.3389/fnsys.2013.00083 24294192 PMC3827539

[B202] Reeb-Sutherland B. C. Fox N. A. (2015). Eyeblink conditioning: a non-invasive biomarker for neurodevelopmental disorders. *J. Autism Dev. Disord.* 45 376–394. 10.1007/s10803-013-1905-9 23942847

[B203] Regier D. A. Kuhl E. A. Kupfer D. J. (2013). The DSM-5: Classification and criteria changes. *World Psychiatry* 12 92–98. 10.1002/wps.20050 23737408 PMC3683251

[B204] Riva D. Annunziata S. Contarino V. Erbetta A. Aquino D. Bulgheroni S. (2013). Gray matter reduction in the vermis and CRUS-II is associated with social and interaction deficits in low-functioning children with autistic spectrum disorders: a VBM-DARTEL Study. *Cerebellum* 12 676–685. 10.1007/s12311-013-0469-8 23572290

[B205] Rivkin M. J. Vajapeyam S. Hutton C. Weiler M. L. Hall E. K. Wolraich D. A. (2003). A functional magnetic resonance imaging study of paced finger tapping in children. *Pediatr. Neurol.* 28 89–95. 10.1016/S0887-8994(02)00492-7 12699857

[B206] Robertson C. E. Baron-Cohen S. (2017). Sensory perception in autism. *Nat. Rev. Neurosci.* 18 671–684. 10.1038/nrn.2017.112 28951611

[B207] Roshani F. Piri R. Malek A. Michel T. M. Vafaee M. S. (2020). Comparison of cognitive flexibility, appropriate risk-taking and reaction time in individuals with and without adult ADHD. *Psychiatry Res.* 284:112494. 10.1016/j.psychres.2019.112494 31439404

[B208] Rumsey J. M. (1985). Conceptual problem-solving in highly verbal, nonretarded autistic men. *J. Autism Dev. Disord.* 15 23–36. 10.1007/BF01837896 3980427

[B209] Russell J. S. R. Horsley V. A. H. (1894). XVIII. Experimental researches into the functions of the cerebellum. *Philos. Trans. R. Soc. Lond.* 185 819–861. 10.1098/rstb.1894.0018 11169987

[B210] Russo N. Flanagan T. Iarocci G. Berringer D. Zelazo P. D. Burack J. A. (2007). Deconstructing executive deficits among persons with autism: implications for cognitive neuroscience. *Brain Cogn.* 65 77–86. 10.1016/j.bandc.2006.04.007 17825970

[B211] Saemundsen E. Juliusson H. Hjaltested S. Gunnarsdottir T. Halldorsdottir T. Hreidarsson S. (2010). Prevalence of autism in an urban population of adults with severe intellectual disabilities–a preliminary study. *J. Intellect. Disabil. Res.* 54 727–735. 10.1111/j.1365-2788.2010.01300.x 20633201

[B212] Sanjeevan T. Cardy R. E. Anagnostou E. (2020). Procedural Sequence Learning in Attention Deficit Hyperactivity Disorder: A Meta-Analysis. *Front. Psychol.* 11:560064. 10.3389/fpsyg.2020.560064 33192824 PMC7655644

[B213] Sarovic D. (2021). A Unifying Theory for Autism: The Pathogenetic Triad as a Theoretical Framework. *Front. Psychiatry* 12:767075. 10.3389/fpsyt.2021.767075 34867553 PMC8637925

[B214] Schellig D. Schuri U. (2009). *n-back verbal.* Moedling: SCHUHFRIED GmbH.

[B215] Schiweck C. Arteaga-Henriquez G. Aichholzer M. Edwin Thanarajah S. Vargas-Cáceres S. Matura S. (2021). Comorbidity of ADHD and adult bipolar disorder: A systematic review and meta-analysis. *Neurosci. Biobehav. Rev.* 124 100–123. 10.1016/j.neubiorev.2021.01.017 33515607

[B216] Schmahmann J. D. (1991). An emerging concept. The cerebellar contribution to higher function. *Arch. Neurol.* 48 1178–1187. 10.1001/archneur.1991.00530230086029 1953406

[B217] Schmahmann J. D. (2019). The cerebellum and cognition. *Neurosci. Lett.* 688 62–75. 10.1016/j.neulet.2018.07.005 29997061

[B218] Schmahmann J. D. Ko R. MacMore J. (2004). The human basis pontis: motor syndromes and topographic organization. *Brain* 127 1269–1291. 10.1093/brain/awh138 15128614

[B219] Schmahmann J. D. Sherman J. C. (1998). The cerebellar cognitive affective syndrome. *Brain* 121 561–579. 10.1093/brain/121.4.561 9577385

[B220] Schreiber H. E. Javorsky D. J. Robinson J. E. Stern R. A. (1999). Rey-Osterrieth Complex Figure performance in adults with attention deficit hyperactivity disorder: a validation study of the Boston Qualitative Scoring System. *Clin. Neuropsychol.* 13 509–520. 10.1076/1385-4046(199911)13:04;1-Y;FT509 10806464

[B221] Schutter D. J. L. G. Enter D. Hoppenbrouwers S. S. (2009). High-frequency repetitive transcranial magnetic stimulation to the cerebellum and implicit processing of happy facial expressions. *J. Psychiatry Neurosci.* 34 60–65. 19125214 PMC2612080

[B222] Sears L. L. Finn P. R. Steinmetz J. E. (1994). Abnormal classical eye-blink conditioning in autism. *J. Autism Dev. Disord.* 24 737–751. 10.1007/BF02172283 7844097

[B223] Sears L. L. Vest C. Mohamed S. Bailey J. Ranson B. J. Piven J. (1999). An MRI study of the basal ganglia in autism. *Prog. Neuropsychopharmacol. Biol. Psychiatry* 23 613–624. 10.1016/S0278-5846(99)00020-2 10390720

[B224] Seidman L. J. Valera E. M. Makris N. (2005). Structural brain imaging of attention-deficit/hyperactivity disorder. *Biol. Psychiatry* 57 1263–1272. 10.1016/j.biopsych.2004.11.019 15949998

[B225] Serra H. G. Duvick L. Zu T. Carlson K. Stevens S. Jorgensen N. (2006). RORalpha-mediated Purkinje cell development determines disease severity in adult SCA1 mice. *Cell* 127 697–708. 10.1016/j.cell.2006.09.036 17110330

[B226] Shakkottai V. G. do Carmo Costa M. Dell’Orco J. M. Sankaranarayanan A. Wulff H. (2011). Early changes in cerebellar physiology accompany motor dysfunction in the polyglutamine disease spinocerebellar ataxia type 3. *J. Neurosci.* 31 13002–13014. 10.1523/JNEUROSCI.2789-11.2011 21900579 PMC3170039

[B227] Sharma S. R. Gonda X. Tarazi F. I. (2018). Autism Spectrum Disorder: Classification, diagnosis and therapy. *Pharmacol. Ther.* 190 91–104. 10.1016/j.pharmthera.2018.05.007 29763648

[B228] Sheppard D. P. Bruineberg J. P. Kretschmer-Trendowicz A. Altgassen M. (2018). Prospective memory in autism: theory and literature review. *Clin. Neuropsychol.* 32 748–782. 10.1080/13854046.2018.1435823 29536800

[B229] Siegel A. Sapru H. N. (2006). *Essential Neuroscience.* Philadelphia, PA: Lippincott Williams & Wilkins.

[B230] Silk T. J. Vance A. Rinehart N. Bradshaw J. L. Cunnington R. (2009). White-matter abnormalities in attention deficit hyperactivity disorder: a diffusion tensor imaging study. *Hum. Brain Mapp.* 30 2757–2765. 10.1002/hbm.20703 19107752 PMC6870653

[B231] Slapik M. Kronemer S. I. Morgan O. Bloes R. Lieberman S. Mandel J. (2019). Visuospatial Organization and Recall in Cerebellar Ataxia. *Cerebellum* 18 33–46. 10.1007/s12311-018-0948-z 29949096

[B232] Sokolovsky N. Cook A. Hunt H. Giunti P. Cipolotti L. (2010). A preliminary characterisation of cognition and social cognition in spinocerebellar ataxia types 2, 1, and 7. *Behav. Neurol.* 23 17–29. 10.1155/2010/395045 20714058 PMC5434399

[B233] Stanley-Cary C. Rinehart N. Tonge B. White O. Fielding J. (2011). Greater disruption to control of voluntary saccades in autistic disorder than Asperger’s disorder: evidence for greater cerebellar involvement in autism? *Cerebellum* 10 70–80. 10.1007/s12311-010-0229-y 21072692

[B234] Steinmetz J. E. (2000). Brain substrates of classical eyeblink conditioning: a highly localized but also distributed system. *Behav. Brain Res.* 110 13–24. 10.1016/S0166-4328(99)00181-3 10802300

[B235] Stezin A. Bhardwaj S. Hegde S. Jain S. Bharath R. D. Saini J. (2021). Cognitive impairment and its neuroimaging correlates in spinocerebellar ataxia 2. *Parkinsonism Relat. Disord.* 85 78–83. 10.1016/j.parkreldis.2021.02.028 33756405 PMC7613150

[B236] Stoodley C. J. (2014). Distinct regions of the cerebellum show gray matter decreases in autism, ADHD, and developmental dyslexia. *Front. Syst. Neurosci.* 8:92. 10.3389/fnsys.2014.00092 24904314 PMC4033133

[B237] Stoodley C. J. (2016). The cerebellum and neurodevelopmental disorders. *Cerebellum* 15 34–37. 10.1007/s12311-015-0715-3 26298473 PMC4811332

[B238] Stoodley C. J. Schmahmann J. D. (2009b). The cerebellum and language: evidence from patients with cerebellar degeneration. *Brain Lang.* 110 149–153. 10.1016/j.bandl.2009.07.006 19664816

[B239] Stoodley C. J. Schmahmann J. D. (2009a). Functional topography in the human cerebellum: a meta-analysis of neuroimaging studies. *Neuroimage* 44 489–501. 10.1016/j.neuroimage.2008.08.039 18835452

[B240] Stoodley C. J. Schmahmann J. D. (2010). Evidence for topographic organization in the cerebellum of motor control versus cognitive and affective processing. *Cortex* 46 831–844. 10.1016/j.cortex.2009.11.008 20152963 PMC2873095

[B241] Stoodley C. J. Valera E. M. Schmahmann J. D. (2010). An fMRI study of intra-individual functional topography in the human cerebellum. *Behav. Neurol.* 23 65–79. 10.1155/2010/840942 20714062 PMC3776583

[B242] Strick P. L. Dum R. P. Fiez J. A. (2009). Cerebellum and nonmotor function. *Annu. Rev. Neurosci.* 32 413–434. 10.1146/annurev.neuro.31.060407.125606 19555291

[B243] Sturm W. (2017). *Manual Perception and Attention Functions.* Mödling: SCHUHFRIED GmbH.

[B244] Styles M. Alsharshani D. Samara M. Alsharshani M. Khattab A. Qoronfleh M. W. (2020). Risk factors, diagnosis, prognosis and treatment of autism. *Front. Biosci.* 25 1682–1717. 10.2741/4873 32472753

[B245] Takeda T. Nakashima Y. Tsuji Y. (2020). Discrepancies in Wechsler Adult Intelligent Scale III profile in adult with and without attention-deficit hyperactivity disorder. *Neuropsychopharmacol. Rep.* 40 166–174. 10.1002/npr2.12106 32333645 PMC7722665

[B246] Tamura I. Takei A. Hamada S. Soma H. Nonaka M. Homma S. (2018). Executive dysfunction in patients with spinocerebellar ataxia type 3. *J. Neurol.* 265 1563–1572. 10.1007/s00415-018-8883-y 29725839

[B247] Tara E. Vitenzon A. Hess E. Khodakhah K. (2018). Aberrant cerebellar Purkinje cell activity as the cause of motor attacks in a mouse model of episodic ataxia type 2. *Dis. Model. Mech.* 11:34181. 10.1242/dmm.034181 30279196 PMC6177005

[B248] Tatar Z. B. Cansız A. (2022). Executive function deficits contribute to poor theory of mind abilities in adults with ADHD. *Appl. Neuropsychol. Adult* 29 244–251. 10.1080/23279095.2020.1736074 32186409

[B249] Teive H. A. G. Arruda W. O. (2009). Cognitive dysfunction in spinocerebellar ataxias. *Dement. Neuropsychol.* 3 180–187. 10.1590/S1980-57642009DN30300002 29213626 PMC5618971

[B250] Ten Brinke M. M. Heiney S. A. Wang X. Proietti-Onori M. Boele H.-J. Bakermans J. (2017). Dynamic modulation of activity in cerebellar nuclei neurons during pavlovian eyeblink conditioning in mice. *eLife* 6:e28132. 10.7554/eLife.28132 29243588 PMC5760204

[B251] Therrien A. S. Bastian A. J. (2015). Cerebellar damage impairs internal predictions for sensory and motor function. *Curr. Opin. Neurobiol.* 33 127–133. 10.1016/j.conb.2015.03.013 25863011 PMC4786071

[B252] Thürling M. Kahl F. Maderwald S. Stefanescu R. M. Schlamann M. Boele H.-J. (2015). Cerebellar cortex and cerebellar nuclei are concomitantly activated during eyeblink conditioning: a 7T fMRI study in humans. *J. Neurosci.* 35 1228–1239. 10.1523/JNEUROSCI.2492-14.2015 25609637 PMC6605546

[B253] Tinius T. P. (2003). The Integrated Visual and Auditory Continuous Performance Test as a neuropsychological measure. *Arch. Clin. Neuropsychol.* 18 439–454. 10.1093/arclin/18.5.439 14591441

[B254] Tucha O. Mecklinger L. Laufkötter R. Kaunzinger I. Paul G. M. Klein H. E. (2005). Clustering and switching on verbal and figural fluency functions in adults with attention deficit hyperactivity disorder. *Cogn. Neuropsychiatry* 10 231–248. 10.1080/13546800444000047 16571461

[B255] Turesky T. K. Olulade O. A. Luetje M. M. Eden G. F. (2018). An fMRI study of finger tapping in children and adults. *Hum. Brain Mapp.* 39 3203–3215. 10.1002/hbm.24070 29611256 PMC6052794

[B256] Uekermann J. Kraemer M. Abdel-Hamid M. Schimmelmann B. G. Hebebrand J. Daum I. (2010). Social cognition in attention-deficit hyperactivity disorder (ADHD). *Neurosci. Biobehav. Rev.* 34 734–743. 10.1016/j.neubiorev.2009.10.009 19857516

[B257] Vaidya C. J. (2011). Neurodevelopmental Abnormalities in ADHD. *Behav. Neurosci. Attent. Deficit Hyperact. Disord. Treat.* 9 49–66. 10.1007/7854_2011_138 21541845 PMC3329889

[B258] van der Horn H. J. Meles S. K. Kok J. G. Vergara V. M. Qi S. Calhoun V. D. (2022). A resting-state fMRI pattern of spinocerebellar ataxia type 3 and comparison with 18F-FDG PET. *Neuroimage Clin.* 34:103023. 10.1016/j.nicl.2022.103023 35489193 PMC9062756

[B259] van Gaalen J. Maas R. P. P. W. M. Ippel E. F. Elting M. W. van Spaendonck-Zwarts K. Y. Vermeer S. (2019). Abnormal eyeblink conditioning is an early marker of cerebellar dysfunction in preclinical SCA3 mutation carriers. *Exp. Brain Res.* 237 427–433. 10.1007/s00221-018-5424-y 30430184 PMC6373441

[B260] Van Overwalle F. Manto M. Cattaneo Z. Clausi S. Ferrari C. Gabrieli J. D. E. (2020). Consensus Paper: Cerebellum and Social Cognition. *Cerebellum* 19 833–868. 10.1007/s12311-020-01155-1 32632709 PMC7588399

[B261] Veddum L. Pedersen H. L. Landert A.-S. L. Bliksted V. (2019). Do patients with high-functioning autism have similar social cognitive deficits as patients with a chronic cause of schizophrenia? *Nord. J. Psychiatry* 73 44–50. 10.1080/08039488.2018.1554697 30636475

[B262] Vik P. W. Cellucci T. Jarchow A. Hedt J. (2004). Cognitive impairment in substance abuse. *Psychiatr. Clin. North Am.* 27 97–109. 10.1016/S0193-953X(03)00110-2 15062633

[B263] Vloet T. D. Neufang S. Herpertz-Dahlmann B. Konrad K. (2006). [Neuroimaging data of ADHD, tic-disorder and obsessive-compulsive-disorder in children and adolescents]. *Z. Kinder Jugendpsychiatr. Psychother.* 34 343–355. 10.1024/1422-4917.34.5.343 16981155

[B264] Voogd J. (2004). “Cerebellum and Precerebellar Nuclei,” in *The Human Nervous System*, eds Paxinos G. Mai J. (San Diego, CA: Elsevier), 10.1016/B978-012547626-3/50012-0

[B265] Voogd J. Glickstein M. (1998). The anatomy of the cerebellum. *Trends Neurosci.* 21 370–375. 10.1016/S0166-2236(98)01318-6 9735944

[B266] Wan N. Chen Z. Wan L. Tang B. Jiang H. (2020). MR Imaging of SCA3/MJD. *Front. Neurosci.* 14:749. 10.3389/fnins.2020.00749 32848545 PMC7417615

[B267] Wang E. Sun M. Tao Y. Gao X. Guo J. Zhao C. (2017a). Attentional selection predicts rapid automatized naming ability in Chinese-speaking children with ADHD. *Sci. Rep.* 7:939. 10.1038/s41598-017-01075-x 28428624 PMC5430513

[B268] Wang Y. Zhang Y.-B. Liu L.-L. Cui J.-F. Wang J. Shum D. H. K. (2017b). A Meta-Analysis of Working Memory Impairments in Autism Spectrum Disorders. *Neuropsychol. Rev.* 27 46–61. 10.1007/s11065-016-9336-y 28102493

[B269] Wang P. Wang J. Jiang Y. Wang Z. Meng C. Castellanos F. X. (2022). Cerebro-cerebellar Dysconnectivity in Children and Adolescents With Attention-Deficit/Hyperactivity Disorder. *J. Am. Acad. Child Adolesc. Psychiatry* 61 1372–1384. 10.1016/j.jaac.2022.03.035 35661770

[B270] Wang S. S.-H. Kloth A. D. Badura A. (2014). The cerebellum, sensitive periods, and autism. *Neuron* 83 518–532. 10.1016/j.neuron.2014.07.016 25102558 PMC4135479

[B271] Wei H. Zou H. Sheikh A. M. Malik M. Dobkin C. Brown W. T. (2011). IL-6 is increased in the cerebellum of autistic brain and alters neural cell adhesion, migration and synaptic formation. *J. Neuroinflammation* 8:52. 10.1186/1742-2094-8-52 21595886 PMC3114764

[B272] Welniarz Q. Worbe Y. Gallea C. (2021). The Forward Model: A Unifying Theory for the Role of the Cerebellum in Motor Control and Sense of Agency. *Front. Syst. Neurosci.* 15:644059. 10.3389/fnsys.2021.644059 33935660 PMC8082178

[B273] Welsh J. P. Oristaglio J. T. (2016). Autism and classical eyeblink conditioning: performance changes of the conditioned response related to autism spectrum disorder diagnosis. *Front. Psychiatry* 7:137. 10.3389/fpsyt.2016.00137 27563293 PMC4980680

[B274] Whitney E. R. Kemper T. L. Bauman M. L. Rosene D. L. Blatt G. J. (2008). Cerebellar Purkinje cells are reduced in a subpopulation of autistic brains: a stereological experiment using calbindin-D28k. *Cerebellum* 7 406–416. 10.1007/s12311-008-0043-y 18587625

[B275] Williams D. L. Minshew N. J. Goldstein G. Mazefsky C. A. (2017). Long-term memory in older children/adolescents and adults with autism spectrum disorder. *Autism Res.* 10 1523–1532. 10.1002/aur.1801 28448695

[B276] Wilson C. E. Happé F. Wheelwright S. J. Ecker C. Lombardo M. V. Johnston P. (2014). The neuropsychology of male adults with high-functioning autism or asperger syndrome. *Autism Res.* 7 568–581. 10.1002/aur.1394 24903974 PMC4489335

[B277] Wing L. Gould J. (1979). Severe impairments of social interaction and associated abnormalities in children: epidemiology and classification. *J. Autism Dev. Disord.* 9 11–29. 10.1007/BF01531288 155684

[B278] Wolff J. J. Jacob S. Elison J. T. (2018). The journey to autism: Insights from neuroimaging studies of infants and toddlers. *Dev. Psychopathol.* 30 479–495. 10.1017/S0954579417000980 28631578 PMC5834406

[B279] Wolpert D. M. Miall R. C. Kawato M. (1998). Internal models in the cerebellum. *Trends Cogn. Sci.* 2 338–347. 10.1016/S1364-6613(98)01221-2 21227230

[B280] Wu X. Liao X. Zhan Y. Cheng C. Shen W. Huang M. (2017). Microstructural Alterations in Asymptomatic and Symptomatic Patients with Spinocerebellar Ataxia Type 3: A Tract-Based Spatial Statistics Study. *Front. Neurol.* 8:714. 10.3389/fneur.2017.00714 29312133 PMC5744430

[B281] Wulff P. Schonewille M. Renzi M. Viltono L. Sassoè-Pognetto M. Badura A. (2009). Synaptic inhibition of Purkinje cells mediates consolidation of vestibulo-cerebellar motor learning. *Nat. Neurosci.* 12 1042–1049. 10.1038/nn.2348 19578381 PMC2718327

[B282] Yap K. H. Abdul Manan H. Yahya N. Azmin S. Mohamed Mukari S. A. Mohamed Ibrahim N. (2022a). Magnetic Resonance Imaging and Its Clinical Correlation in Spinocerebellar Ataxia Type 3: A Systematic Review. *Front. Neurosci.* 16:859651. 10.3389/fnins.2022.859651 35757531 PMC9226753

[B283] Yap K. H. Kessels R. P. C. Azmin S. van de Warrenburg B. Mohamed Ibrahim N. (2022b). Neurocognitive Changes in Spinocerebellar Ataxia Type 3: A Systematic Review with a Narrative Design. *Cerebellum* 21 314–327. 10.1007/s12311-021-01282-3 34231180

[B284] Young S. Morris R. Toone B. Tyson C. (2007). Planning ability in adults with attention-deficit/hyperactivity disorder. *Neuropsychology* 21 581–589. 10.1037/0894-4105.21.5.581 17784806

[B285] Yuan H.-L. Lai C. Y. Y. Wong M. N. K. Kwong T. C. Choy Y. S. Mung S. W. Y. (2022). Interventions for Sensory Over-Responsivity in Individuals with Autism Spectrum Disorder: A Narrative Review. *Children* 9:101584. 10.3390/children9101584 36291519 PMC9601143

[B286] Zawacki T.M. Grace J. Friedman J. H. Sudarsky L. (2002). Executive and emotional dysfunction in Machado-Joseph disease. *Mov. Disord.* 17 1004–1010. 10.1002/mds.10033 12360550

[B287] Zwick G. P. (2017). Neuropsychological assessment in autism spectrum disorder and related conditions. *Dialogues Clin. Neurosci.* 19 373–379. 10.31887/DCNS.2017.19.4/gzwick 29398932 PMC5789214

