# Corrigendum: Neurocognitive and cerebellar function in ADHD, autism and spinocerebellar ataxia

**DOI:** 10.3389/fnsys.2024.1462062

**Published:** 2024-08-20

**Authors:** Maurizio Cundari, Susanna Vestberg, Peik Gustafsson, Sorina Gorcenco, Anders Rasmussen

**Affiliations:** ^1^Department of Experimental Medical Science, Faculty of Medicine, Lund University, Lund, Sweden; ^2^Unit of Neuropsychiatry, Hospital of Helsingborg, Helsingborg, Sweden; ^3^Unit of Neurology, Hospital of Helsingborg, Helsingborg, Sweden; ^4^Department of Psychology, Faculty of Social Science, Lund University, Lund, Sweden; ^5^Child and Adolescent Psychiatry, Department of Clinical Sciences Lund, Medical Faculty, Lund University, Lund, Sweden; ^6^Department for Clinical Sciences Lund, Neurology, Lund University, Skåne University Hospital, Lund, Sweden

**Keywords:** cerebellum, cognition, neuropsychology, neurology, neuropsychiatry, spinocerebellar ataxia (SCA), attention deficit hyperactivity disorder (ADHD), autism spectrum disorder (ASD)

In the published article, there was an error in Table 1 as published, where under the column ASD – Naming ability shows 3 arrows $\begin{array}{c} ↓\\ \begin{array}{c} ↓↓ \end{array} \end{array}$.

The correct Table 1 under the column ASD – Naming ability should have only one arrow ↓. The corrected version of Table 1 appears below.

**Table 1:** Summarizes the evidence in the papers reviewed.

**Cognitive domain**		**SCA3**	**ADHD**	**ASD**
Executive functions	Inhibition	↓↓	↓↓	↓
	Shifting	↓	↓↓	↓
	Auditory working memory	↓	↓⇄	↓↓
	Visuospatial working memory	↓	↓⇄	↓
Attention and processing speed	Complex attention	↓⇄	↓↓	↓
	Processing Speed	↓⇄	↓↓	↓⇄
Language	Semantic fluency	↓⇄	⇄	↓
	Phonemic fluency	↓	⇄	↓
	Category switching	↓	⇄	↓
	Naming ability	↓	↓	↓
	Verbal intelligence quotient	↓⇄	↓	↓↓
Memory	Verbal immediate recall	↓⇄	↓	↓⇄
	Verbal delayed recall	↓↓	↓↓	↓
	Verbal recognition	↓⇄		↓
	Visual immediate recall	↓	↓	↓
	Visual delayed recall	↓	⇄	↓
	Visual recognition	⇄		↓
	Procedural memory	↓⇄	⇄	↓
Visuospatial perception and visuospatial ability	Visuospatial perception	↓⇄	↓⇄	⇄
	Visuospatial ability	↓⇄	↓⇄	⇄
	Perceptual quotient	↓⇄		
Social cognition	Theory of mind	↓	↓⇄	↓↓

The symbols indicate to what extent SCA3, ADHD, and ASD are associated with deficits in different cognitive domains. The table also indicates whether is a consensus or whether the papers reviewed have different findings. ↓↓ = strong evidence of impairments: (multiple studies converging on the same conclusion). ↓ = weak evidence of impairment (previous studies have low number of subjects and multiple confounders which effect the study results). ⇄ = contrasting results (results in previous studies show that cognitive domain was impaired in some studies but intact in other studies).

In the published article, there was an error in **Attention deficit hyperactivity disorder**, *Summary*, paragraph 3, where the word “(transfer?)” was incorrectly included in brackets when it should have replaced the word “store.” The corrected sentence appears below:

“Hence, the ability to transfer information to the declarative long-term memory is not affected, but rather the retrieval of information.”

The authors apologize for these errors and state that they do not change the scientific conclusions of the article in any way. The original article has been updated.

